# Metastable silica high pressure polymorphs as structural proxies of deep Earth silicate melts

**DOI:** 10.1038/s41467-018-07265-z

**Published:** 2018-11-15

**Authors:** E. Bykova, M. Bykov, A. Černok, J. Tidholm, S. I. Simak, O. Hellman, M. P. Belov, I. A. Abrikosov, H.-P. Liermann, M. Hanfland, V. B. Prakapenka, C. Prescher, N. Dubrovinskaia, L. Dubrovinsky

**Affiliations:** 1 Photon Sciences, Deutsches Elektronen-Synchrotron (DESY), Notkestraße 85, 22607 Hamburg, Germany; 2 Bayerisches Geoinstitut, University of Bayreuth, Universitätsstraße 30, 95440 Bayreuth, Germany; 3 Materials Modeling and Development Laboratory, National University of Science and Technology ‘MISIS’, Leninsky Avenue 4, 119049 Moscow, Russia; 4 Department of Physics, Chemistry and Biology, Linköping University, SE-581 83 Linköping, Sweden; 5 Department of Applied Physics and Materials Science, California Institute of Technology, 1200 East California Boulevard, Pasadena, California 91125 USA; 6 European Synchrotron Radiation Facility (ESRF), 6 Rue Jules Horowitz, 38000 Grenoble, France; 7 Center for Advanced Radiation Sources, University of Chicago, 5640 South Ellis Avenue, Chicago, Illinois 60637 USA; 8 Material Physics and Technology at Extreme Conditions, Laboratory of Crystallography, University of Bayreuth, Universitätsstraße 30, 95440 Bayreuth, Germany; 9 Present Address: School of Physical Sciences, The Open University, Walton Hall, Milton Keynes, MK7 6AA UK; 10 Present Address: Institute of Geology and Mineralogy, Universität zu Köln, Zülpicher Straße 49b, 50674 Köln, Germany

## Abstract

Modelling of processes involving deep Earth liquids requires information on their structures and compression mechanisms. However, knowledge of the local structures of silicates and silica (SiO_2_) melts at deep mantle conditions and of their densification mechanisms is still limited. Here we report the synthesis and characterization of metastable high-pressure silica phases, coesite-IV and coesite-V, using in situ single-crystal X-ray diffraction and ab initio simulations. Their crystal structures are drastically different from any previously considered models, but explain well features of pair-distribution functions of highly densified silica glass and molten basalt at high pressure. Built of four, five-, and six-coordinated silicon, coesite-IV and coesite-V contain SiO_6_ octahedra, which, at odds with 3^rd^ Pauling’s rule, are connected through common faces. Our results suggest that possible silicate liquids in Earth’s lower mantle may have complex structures making them more compressible than previously supposed.

## Abstract (web summary)

As silica melts are believed to be important components of the Earth’s mantle, their structure should determine many of its properties. Here, the authors identify two crystalline modifications of SiO_2_, whose local structures closely resemble those of known melts, providing a structural model for their atomic ordering at the nanoscale.

## Introduction

There is compiling evidence that neutrally or negatively buoyant silicate melts are present at the base of the Earth’s mantle^1–6^. Knowledge on physical and chemical properties of the melts is important for understanding evolution of the deep Earth interiors. The local structure of melts, which is roughly characterized by the coordination number of silicon atoms and the way how the silicon polyhedra are interconnected at certain pressure–temperature conditions, can be modeled using molecular dynamics simulations^7,8^, Bader’s atoms-in-molecules approach^9^, or studied experimentally on silica or silicate glasses^10–12^. Recent experimental studies of silica glass suggest that the coordination number of silicon atoms drastically increases from 4 to 6 between 15 and 60 GPa and then, up to ~100 GPa, it is either constant^13^ or increases^11^ to 7. However, structural models are lacking and the interpretation of observations remains ambiguous, as the existence of penta-coordinated silicon in glass remains elusive. A very convincing method to obtain a structural model of noncrystalline silica material is to compare (or fit) experimental total scattering data with the pair-distribution function (PDF) of known crystalline phase(s)^14^. In this manner, Keen and Dove^14^ determined that the local structure of silica glass at ambient conditions has strong similarities with HP-tridymite and β-cristobalite. So far, however, there were no crystalline phase(s) which could describe features of total scattering data of silica and/or silicate glasses at high pressure.

**Fig. 1:**
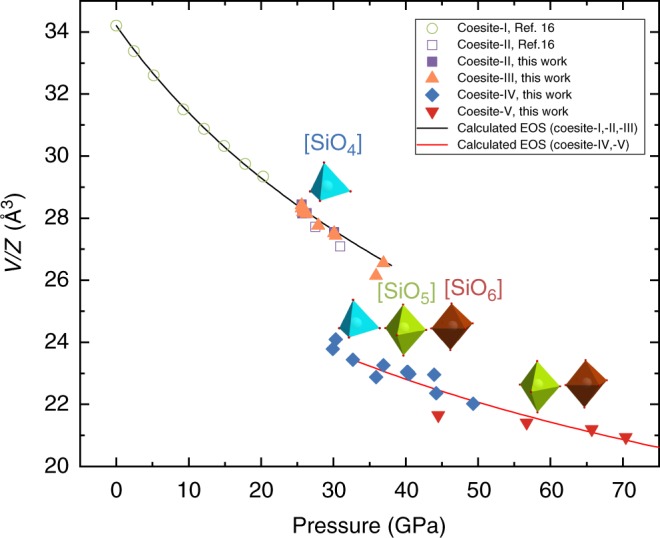
The *P*–*V* data of squeezed coesite. Unit cell volumes are normalized to the number of formula units *Z*. Open symbols represent literature data^15,16^ (in the present work, we used samples of the same coesite which was studied in refs.^15,16^). The black solid line represents a fit of all the *VP*-data for coesite-I, II, and III with the Birch–Murnaghan equation of state (EOS) (*V*_0_/*Z* = 34.20(1) Å^3^, *K*_0_ = 103(2) GPa, and *K*´ = 3.02(15)). Combined pressure–volume data for coesite-IV and coesite-V were fitted with the second-order Birch–Murnaghan equation of state (*V*_32.7_/*Z* = 23.44(3) Å^3^, *K*_32.7_ = 254(9) GPa) and are shown on the graph by a solid red line. Colored polyhedra indicate the building blocks characteristic for the structures of silica polymorphs in the corresponding pressure regions. Uncertainties in the unit cell volumes are less than the symbol sizes and are therefore not shown. As seen, coesite-III and coesite-IV show significant scatter in values of the unit cell volumes that might be attributed to different deviatoric stresses in samples from different runs

Coesite, a dense silica polymorph, remains a subject of intense studies at high pressures and variable temperatures. At ambient conditions, coesite has a monoclinic crystal structure (space group *C*2/*c*, further called “coesite-I”) and above ∼20 GPa, it undergoes a phase transition with doubling of the unit cell parameter *b* (space group *P*2_1_/*c*, “coesite-II”)^15,16^. Like in coesite-I, all silicon atoms in coesite-II were found in SiO_4_ tetrahedra linked through vertexes (Fig. 1, Supplementary Fig. 1a, b). Above 31 GPa, coesite-II transforms into a phase tentatively indexed as triclinic (“coesite-III”)^16^. On the contrary, based on a combination of X-ray diffraction and ab initio metadynamics simulation, Hu et al.^17^ have proposed a transition between 26 and 53 GPa from coesite-I to post stishovite (i.e., built of only SiO_6_ octahedra) through a series of triclinic intermediate phases featuring both SiO_4_ tetrahedra and SiO_6_ octahedra. Further first-principles calculations made by Liu et al.^18^ suggest that post-stishovite phase should eventually transform to the one with α-PbO_2_ structure. So far, the structures of coesite-III and other possible high-pressure polymorphs of coesite remain unknown calling for further investigations.

Here, we apply single-crystal X-ray diffraction in diamond anvil cells (see Methods) in order to study SiO_2_ phases, which appear on compression of coesite, and their high-pressure behavior. Several independent experiments are performed at different synchrotron radiation facilities at pressures over 70 GPa. The results are summarized in Supplementary Table 1. We show that high-pressure phases of coesite can be used as proxies of the local structure of high-pressure silica melts. The crystal structure of coesite’s high-pressure phases can also give an insight into the mechanisms of silica glass densification.

## Results

### Crystal structure of coesite-III

In agreement with previous data, in all experiments, we observed the transformation of coesite-I to coesite-II at pressures exceeding 20 GPa (Supplementary Figs. 1, 2). A close examination of the diffraction patterns collected just above 25 GPa revealed a set of reflections which did not belong to coesite-II (Supplementary Fig. 2), and therefore should be attributed to a different phase. Indexed as triclinic (space group *P*-1), it turned out to be coesite-III (Supplementary Tables 2–4, Supplementary Figs. 1, 2).

We solved and refined the crystal structure of coesite-III using a dataset collected at 28 GPa (Supplementary Figs. 1, 2, Supplementary Tables 3, 4, Supplementary Data 1). Silicon atoms in coesite-III (Supplementary Fig. 1) occupy oxygen tetrahedra linked together through common vertices. Similar to coesite-I and coesite-II, the major building blocks of the structure are four-membered rings of SiO_4_ tetrahedra (Supplementary Fig. 1). Phase transitions to coesite-II and coesite-III have a minor effect on the molar volume (Fig. 1), so that the compressional behavior of all coesite phases with tetra-coordinated silicon may be described by the same Birch–Murnaghan equation of state (EOS) with *V*_0_/*Z* = 34.20(1) Å^3^, *K*_0_ = 103(2) GPa, and *K*´ = 3.02(15).

### Crystal structures of coesite-IV and coesite-V

On compression beyond ca. 30 GPa, a new set of reflections (Supplementary Fig. 2) manifest the presence of a new triclinic (space group *P*-1) phase, which we called coesite-IV (Supplementary Table 2). Upon further compression, the reflections of coesite-IV split and a new triclinic (space group *P*-1) phase, coesite-V emerged. Above ~50 GPa, only coesite-V was found (Fig. 1). Although upon compression the quality of coesite-V crystals deteriorates, the phase remains crystalline (Supplementary Fig. 2) at ambient temperature to at least 70 GPa (highest pressure achieved in this study).

**Fig. 2:**
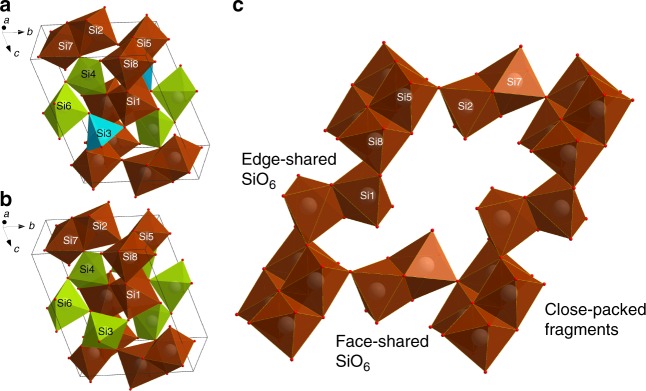
Crystal structures of coesite-IV and coesite-V. Polyhedral models of the structures of coesite-IV (**a**) and coesite-V (**b**) (SiO_6_ octahedra are brown; SiO_5_ polyhedra are green; SiO_4_ tetrahedra are blue); a fragment of the structures, similar for both coesite-IV and coesite-V, showing a three-dimensional framework of SiO_6_ octahedra (**c**)

The structures of coesite-IV and of coesite-V were solved and refined using the datasets collected at ~36, 40, 44, and 49 GPa for the former (Supplementary Tables 5–8, Supplementary Data 2–5), and at ∼57 GPa for the latter (Supplementary Data 6) (see also Supplementary Tables 2, 3, 9). For silica, known for its rich polymorphism^17^, the structures of coesite-IV and coesite-V are unusually complex (Fig. 2), but very alike. Their lattice parameters are very similar, and the unit cells contain 16 SiO_2_ formula units. The structure of coesite-IV possesses *tetra-*, *penta-*, and *hexa-*coordinated silicon; the structure of coesite-V maintains only *penta-* and *hexa-*coordinated silicon (Supplementary Fig. 3). In fact, analyzing them in detail, one can see that the structures of coesite-IV and coesite-V may be considered as a three-dimensional framework of face- and edge-sharing octahedra with the empty space, filled by SiO_5_ and SiO_4_ (in coesite-IV), or only SiO_5_ (in coesite-V) polyhedra (Fig. 2). The findings described above present three crystalchemical surprises at once: *penta-*coordinated silicon, face-sharing SiO_6_ octahedra, and the number of essentially different constituents in the structure of silica. These results are discussed below.

The size and shape of SiO_4_ tetrahedra in coesite-IV (Fig. 2) are comparable with those in coesite-III (Supplementary Fig. 1), although the tetrahedra in coesite-IV are more distorted in terms of the bond angle variance (BAV)^19^. Measured in squared degrees, BAV in coesite-IV is equal to ∼81, whereas it is below ∼63 in coesite-III.

Although penta-coordinated silicon was previously observed in silicates^20–24^, coesite-IV and coesite-V are the first experimentally observed silica polymorphs featuring SiO_5_ polyhedra. There are two crystallographically distinctive penta-coordinated silicon atoms (Si4 and Si6) in coesite-IV and three (Si3, Si4, and Si6) in coesite-V (Fig. 2). Although the range of Si–O bond lengths and their average are similar for all SiO_5_ polyhedra, the shape of the polyhedra varies considerably (Fig. 2), accommodating features common for a trigonal bipyramid and for a square pyramid.

The SiO_6_ octahedra in both coesite-IV and coesite-V demonstrate a considerable variation in the volume (Supplementary Fig. 4a) and Si–O distances (Supplementary Fig. 4b), but they are comparable with those in stishovite, the only known silica phase with *hexa-*coordinated silicon whose structure has been reliably and systematically studied experimentally at high pressures^25^. However, the distance between atoms Si2 and Si7 is unexpectedly short (∼2.44 Å). It is by ~8% shorter than could be anticipated for the structure with the *hexa-*coordinated silicon at ∼50 GPa (Supplementary Fig. 4c). The reason is the highly unusual type of connection of (Si2)O_6_ and (Si7)O_6_ octahedra—through the common face (Fig. 2). Such a structural element has been neither experimentally observed for any silicon compounds, nor expected for small and high-valence cations like Si^4+^.

In coordinated structures, the occurrence of polyhedra sharing edges or faces is only common for large cations, but very rarely seen for high-valence and low-coordinated (with the coordination number six or lower) small cations. This is the main thrust of third Pauling’s rule^26,27^, which is especially strict for compounds such as borates, phosphates, and silicates^27^. Coesite-IV and coesite-V represent the first experimental examples of violation of third Pauling’s rule for SiO_6_ octahedra linked through faces. In fact, structures of coesite-IV and coesite-V do not obey also fifth Pauling’s rule (“the rule of parsimony”)^26,27^. This rule suggests that the number of essentially different kinds of constituents forming a crystal structure tends to be small. Although silicates provide many exceptions from the rule (e.g., the structure of MgSiO_3_ garnet features both silicon tetrahedra and octahedra), all hitherto-known silica polymorphs^15,16,28,29^ (at least 30 different phases) are built either of tetrahedra or octahedra solely. Thus, coesite-IV and coesite-V are the first experimentally observed silica polymorphs built of polyhedra of different types.

It should be noted that the complex structure of silica and silicate liquids under compression has been proposed previously. First-principles molecular dynamic simulations on silica liquid^7^ suggest that at high compression, with volume contraction *V*/*V*_0_ < 0.7, which corresponds to compression of coesite beyond 30 GPa (i.e., exactly the pressure at which a transition to coesite-IV occurs), silicon may simultaneously adopt several types of coordination, namely five-, six-, and sevenfold, with the coordination polyhedra to be significantly distorted. Face-sharing of the polyhedra was also suggested, but the kind of the face-sharing was not explicitly specified^7^. Similar results were obtained in molecular dynamic simulations for MgSiO_3_ glass^8^.

### Stability of coesite-IV and coesite-V

A phase with a crystal structure disobeying Pauling’s rules is expected to be metastable. Ab initio simulations (see Methods) confirm that both coesite-IV and coesite-V are dynamically stable at the pressures where they were observed experimentally (Supplementary Fig. 5). At lower pressure, coesite-IV is more stable than coesite-V (Supplementary Fig. 5, inset), in agreement with the experiment. Calculated equations of state (Supplementary Fig. 6) and lattice parameters (Supplementary Table 10) of the two phases are in very good agreement with experimental data as well. With increasing pressure, coesite-IV becomes less stable thermodynamically and dynamically, to the extent that it transforms into coesite-V phase at pressures above ~50 GPa without a barrier (Supplementary Movie 1). At the same time, both phases are energetically highly unfavorable—at 38 GPa, where coesite-V and coesite-IV are nearly degenerate in enthalpy in our theoretical calculations, the calculated enthalpy difference between them and the ground state is 0.389 eV·ion^−1^ (Supplementary Fig. 5, inset). In fact, the very large differences in enthalpy between phases stable at corresponding conditions (stishovite, CaCl_2_-type, seifertite) and coesite-IV, V indicate that using modern algorithms of crystal structure predictions or molecular dynamic simulations^18,30,31^, it would be highly difficult (if possible at all) to envisage their existence. On the other hand, the observed enthalpy difference is similar to the values calculated for carbon polymorphs, for example, such as diamond and C_60_^32^.

### Notes on the compression mechanism of coesite

All known high-pressure silica phases with six-coordinated silicon are constructed on the basis of distorted hexagonal close-packing (hcp) of oxygen atoms^27–29,33^. Pressurization of low-density silica phases (like cristobalite or tridymite), which already contain distorted/defect close-packed oxygen layers^29,34^, results in their transformations into dense phases, built of SiO_6_–octahedra^35,36^. This happens relatively easily, in a moderate pressure range of ∼10 – 30 GPa, as far as within the close-packed oxygen arrays there are only tetrahedral and octahedral interstices, which can be occupied by the cations without severe distortions of the framework. Pressurization of coesite leads to a different result, because its structure does not provide an easy way for the formation of a total hcp framework of oxygen atoms. Although in coesite-IV and coesite-V, one can see fragments closely resembling connections of octahedra in close-packed structures^33,34,36^ (Fig. 2), these fragments do not form continuous layers, and silicon may locate in five-coordinated sites. This picture qualitatively agrees with the changes observed upon the transition from coesite-IV to coesite-V (Fig. 2, Supplementary Fig. 3): (Si3)O_4_–tetrahedra in coesite-IV turn into (Si3)O_5_polyhedra in coesite-V. It was suggested long time ago and by now generally accepted^37^ that silica and silicate structures are based on close-packing due to a relatively high ionicity of the Si–O bond. One could hypothesize that the appearance of an unusual structural element (penta-coordinated silicon, octahedra sharing faces) may be related to increased covalency of Si–O bonding with pressure. However, Bader analysis of charge variation in different silica phases with pressure (Supplementary Fig. 7) shows that this is not the case. Our results suggest that penta-coordinated silicon may be a usual component of the intermediate structures or metastable phases upon compression of silicates with oxygen arrays significantly deviated from close-packing (e.g., tectosilicates with large cations, borosilicates, and others).

### Comparison with PDFs of silica glass and molten basalt

**Fig. 3:**
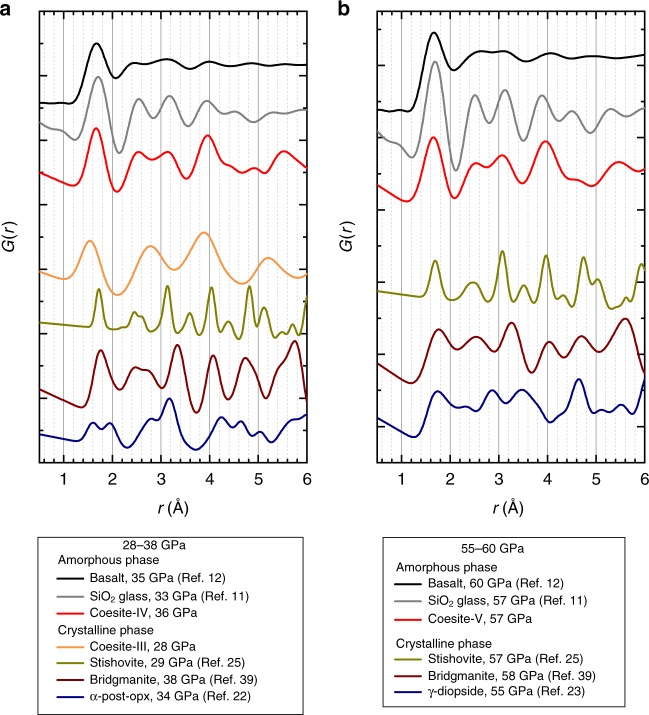
Pair-distribution functions of silica and silicate phases. Solid curves represent pair-distribution functions calculated for silica polymorphs (this work and ref. ^25^) and silicates^22, 23, 39^, compared with those for basalt^12^ and silica^11^ glass measured at different pressures: **a**, in the range from 28 to 38 GPa; **b**, in the range from 55 to 60 GPa

In Fig. 3 (see also Supplementary Fig. 8), we compare pair-distribution functions calculated for different silica polymorphs^25,38^ (including different coesite-derived phases) and silicates^22,23,39^ with the experimental data measured for silica glass^11^ and basalt^12^, as a function of pressure (see Methods). The PDF of silica glass at low pressure has similarities with the PDFs of crystalline silica phases with tetra-coordinated silicon (close positions of the first four peaks), like coesite-I and especially cristobalite. At pressures around or above 30 GPa, the silica glass PDF changes considerably; it does not look anymore as from phases containing only SiO_4_ units (like coesite-III), or only octahedra (like stishovite). On the contrary, there are striking similarities in the PDF of glass at 33 GPa and of coesite-IV at about the same pressure (Fig. 3a). At higher pressures, at about 60 GPa, the PDF of glass remains to be alike of the PDF of coesite-V containing 3/8 of silicon atoms penta-coordinated, and 5/8—in SiO_6_ octahedra (Fig. 3b). The PDFs of molten basalt^12^ at ~30 and ~60 GPa possess the same features (maxima and minima) as PDFs of coesite-IV and coesite-V at corresponding pressures. Reported recently^40^, PDFs of MgSiO_3_ glass up to about 110 GPa are significantly different from that of molten basalt^12^, suggesting that compressed pure MgSiO_3_ glass cannot represent mantle-related liquids. Densities of coesite-IV and coesite-V at a pressure above ∼45 GPa within the uncertainty of measurements coincide with the density of silica glass^10^ (Supplementary Fig. 9a). These observations are arguments in support of analogous atomic arrangements in compressed silica glass and in coesite-IV or coesite-V. Our results also indicate that (a) silica glass indeed contains SiO_5_ polyhedra (and not just a mixture of tetrahedra and octahedra in a certain proportion), and (b) in silica glass, a transition from 4- to 6- coordination may not be over at 60–70 GPa, as suggested previously^10,41^.

## Discussion

Our observations imply a possible formation of a very complex network of polyhedra, including face-shared octahedra in silicate liquids at high pressures. A possible influence of such structural peculiarities on the elastic properties of silica (and indirectly on silicate melts) (Supplementary Fig. 9; Supplementary Table 11) may be assessed through a comparison of the compressibility and bulk sound velocities of coesite-IV and coesite-V with those of dense silica phases with hexa-coordinated silicon (stishovite, CaCl_2_-structured SiO_2_, and seifertite)^42^. Obviously, the presence of unconventional structural elements like SiO_5_ polyhedra and face-sharing octahedra significantly decreases density and, what is even more important, it leads to a drop in the bulk sound velocity above 40 GPa (by about 10% compared with high-pressure crystalline SiO_2_ phases; Supplementary Fig. 9c). If silicate liquids with such properties are present in the lower mantle, they should be clearly seismically detectable.

## Methods

### Sample preparation

The starting material for the coesite synthesis was SiO_2_ glass powder with very low trace-element content, as analyzed at the BGI using LA-ICP-MS: Al 20 ppm, Ge 1.3 ppm, Na 1.0 ppm, Li 0.8 ppm, and B, Ti, Fe, Ga, Rb, and Sn below the detection limits. Coesite single crystals were synthesized by mixing the starting powder with ~5 wt% distilled water inside a platinum capsule, which was then welded shut. The capsule was first placed into pyrophyllite sleeves and then in a 0.5′′ talc-pyrex piston-cylinder assembly containing internal, tapered graphite resistance furnaces. The mixture was pressurized to 3.5 GPa and slowly heated up to 1250 °C, kept at this temperature for ~15 h, then cooled down to 1100 °C in 5 h, and finally quenched. Slow cooling procedure and water-saturated conditions resulted in growth of relatively large (above 100 μm in linear dimensions) crystals. No Raman peaks were observed in the spectra of synthesized coesite in the O–H vibration region (2800–3400 cm^–1^).

Single crystals of coesite with an average size of 0.02 × 0.02 × 0.005 mm^3^ were preselected on a three-circle Bruker diffractometer equipped with a SMART APEX CCD detector and a high-brilliance Rigaku rotating anode (Rotor Flex FR-D, Mo-*K*α radiation) with Osmic focusing X-ray optics.

### X-ray diffraction

The single-crystal XRD experiments were conducted on the Extreme Conditions Beamline P02.2 at PETRA III, Hamburg, Germany (MAR345dtb image plate detector, Perkin Elmer XRD1621 flat panel detector, λ = 0.2898–0.2902 Å); on the ID09A (now ID15B) beamline at the European Synchrotron Radiation Facility (ESRF), Grenoble, France (MAR555 detector, λ = 0.41273 Å); and on the 13-IDD beamline at the Advanced Photon Source (APS), Chicago, USA (MAR165 CCD detector, λ = 0.3344 Å). The X-ray spot size depended on the beamline settings and varied from 4 to 30 μm. Sample-to-detector distance, coordinates of the beam center, tilt angle, and tilt plane rotation angle of the detector images were calibrated using CeO_2_ (for data collected at P02.2 beamline), Si (ID09A), and LaB_6_ (13-IDD) powders. XRD images were collected during continuous rotation of DACs typically from –20 to + 20 on omega; while data collection experiments were performed by narrow 0.5–1° scanning of the same omega range. DIOPTAS software^43^ was used for preliminary analysis of the 2D images and calculation of pressure values from the positions of the XRD lines of Ne.

Three sets of experiments were performed. In each experiment, two single crystals of coesite together with a small ruby chip (for pressure estimation) were loaded into BX90-type DACs^44^. Neon was used both as a pressure-transmitting medium and as a pressure standard in all experiments. Neon was loaded with a gas-loading system installed at the Bayerisches Geoinstitut^45^.

The first DAC was gradually compressed to ~57 GPa, while the single-crystal XRD has been measured only for one crystal at five selected pressure points (namely at 5.8(5), 27.9(5), 35.9(7), 44.5(5), and 57.1(6) GPa). Then, the cell was decompressed with ~5–10 GPa pressure step, and due to sample deterioration, only wide images were measured.

In the second DAC, single-crystal XRD has been measured at six selected pressure points (namely at 14.2(3), 21.6(4), 27.5(3), 29.9(6), 32.7(5), and 43.8(5) GPa) for the first crystal and at two pressure points for another one (27.9(4) and 43.9(4) GPa).

In the third run, DAC was compressed to 70 GPa, single-crystal XRD has been measured at 5.3(5), 25.6(3), 30.3(6), 36.9(4), 40.3(5), 44.2(4), 49.3(8), 56.8(9), 65.7(9), and 70.4(9) GPa for the first crystal, and at 5.3(5), 26.3(4), and 30.1(4) GPa for the second one. At selected pressure points, single-crystal XRD was collected in two orientations of the DAC in order to increase data completeness.

It should be noted that only six measurements resulted in successful structure solution and satisfactory refinement (one for coesite-III, four for coesite-IV, and one for coesite-V). The reasons are close peaks overlapping since the samples often contained two phases, early sample deterioration under compression which resulted in peak broadening and low intensity of the reflections.

The detailed summary of the experiments performed, together with determined phase compositions are summarized in Supplementary Table 1. Unit cell parameters of the observed phases are given in Supplementary Table 2. Details of crystal structure refinements of SiO_2_ high-pressure phases are given in Supplementary Table 3. Supplementary Tables 4–9 contain information on atomic coordinates and equivalent isotropic displacement parameters of high-pressure coesite phases.

Processing of XRD data (the unit cell determination and integration of the reflection intensities) was performed using CrysAlisPro software^46^. Indexing of the unit cell was performed on about 50 reflections manually selected in the reciprocal space viewer (Ewald explorer implemented in CrysAlisPro software). The reflections were selected in order to follow a 3D lattice in the reciprocal space. Then the found unit cell was refined on the whole set of the reflections with 0.05 tolerance (maximum allowed displacement of the *h*,*k*,*l* indices from an integer). Empirical absorption correction was applied using spherical harmonics, implemented in the SCALE3 ABSPACK scaling algorithm, which is included in the CrysAlisPro software. A single crystal of an orthoenstatite ((Mg_1.93_,Fe_0.06_)(Si_1.93_,Al_0.06_)O_6_, *Pbca*, *a* = 8.8117(2), *b* = 5.18320(10), and *c* = 18.2391(3) Å), was used to calibrate the instrument model of CrysAlisPro software (sample-to-detector distance, the detector’s origin, offsets of the goniometer angles, and rotation of the X-ray beam and the detector around the instrument axis).

### Structure solution and refinement of coesite-III

In the experimental datasets, coesite-III was often found together with either coesite-II or coesite-IV. When coesite-II and coesite-III are found together, orientations of their b* axes coincide, implying that lattice planes (0 1 0) of the two crystals should be parallel to each other. In the case of coesite-III and coesite-IV, we observed that the reciprocal plane a*b* of coesite-III coincides with the a*c* plane of coesite-IV. Then the ***c*** lattice vector of coesite-III and the ***b*** lattice vector of coesite-IV should be colinear.

The crystal structure of coesite-III was refined only at 27.9(5) GPa, due to weakness of the diffraction data in other datasets. At this pressure point, no additional phases (coesite-II or coesite-IV) were found, but instead several domains of coesite-III were observed. The number of overlapped peaks between the most two intense domains did not exceed 20% of the total peak number. The second domain is rotated relative to the first one by ~178.8° about the b* axis. The crystal structure of coesite-III was solved using the data from the most intense domain.

In the experiment, we collected 1295 reflections, which were merged based on the crystal symmetry to 833 independent reflections with *R*_int_ = 3.29%. At *d* = 0.8 Å, completeness of the data was 25.3%. The structure was determined by a direct method using SHELXS^47^ software. After the structure solution, most of the atoms were found and the rest of them were located from a series of difference Fourier map cycles. The crystal structure was refined against *F*^2^ on all data by full-matrix least squares with the SHELXL^47^ software. The amount of the collected data allowed us to refine the structure only in an isotropic approximation. The resulting ratio between data (833) and the number of parameters (143) was ~6. Five reflections were omitted from the refinement due to their overlap with diamond peaks. The final structure converged with *R*_1_ = 14.81%, *wR*_2_ = 36.32%, and GOF = 1.625 for all 833 unique reflections [*R*_1_ = 13.64%, *wR*_2_ = 34.72%, for those 681 data with *I* > 2σ(*I*)]. On a final difference Fourier map, we observed no peaks higher than 1.4 e·Å^−2^. The most of the intense peaks were within 1 Å of the Si atoms, but several peaks of about 1 e·Å^−2^ were found nearby oxygen atoms (1.1–1.2 Å) probably due to the twinning present. Values of thermal parameters of all atoms except O(5) are within the normal interval (0.015–0.027 A^2^). Atom O(5) is located on a special position (0 0 0.5) and has a larger thermal parameter (0.05 A^2^) probably due to the twinning present or even lower symmetry (*P*1) of the structure.

### Structure solution and refinement of coesite-IV

Coesite-IV has a triclinic symmetry, and data completeness of a single dataset was not enough to solve the crystal structure. In order to increase data completeness, we created a combined reflection file from two *hkl*-files (obtained from datasets collected at 29.9(6) and 40.2(7) GPa) using program XPREP^48^. Then the structure could be successfully determined by the direct method using SHELXS^47^ software. After the structure solution, most of the atoms were found and the rest of them were located from a series of difference Fourier map cycles. The obtained model was used for the refinement of the structure in the individual datasets. The crystal structure was refined against *F*^2^ on all data by full-matrix least squares with the SHELXL^47^ software. The crystal structure of coesite-IV was refined at 35.9(7), 40.2(7), 44.2(4), and 49.3(8) GPa. At the other pressure points, the structure refinement was not possible due to weakness of the diffraction data.

In the experiment at 35.9(7) GPa, we collected 678 reflections, which were merged based on the crystal symmetry to 452 independent reflections with *R*_int_ = 5.49. At *d* = 0.8 Å, completeness of the data was 25.5%. The amount of the collected data allowed us to refine the structure only in an isotropic approximation. The resulting ratio of the number of reflections (452) and the number of parameters (97) was ~5. Two reflections were omitted from the refinement due to overlap with the diamond peaks. The final structure was refined to convergence with *R*_1_ = 10.86%, *wR*_2_ = 22.80%, and GOF = 1.122 for all 452 unique reflections [*R*_1_ = 8.11%, *wR*_2_ = 20.83%, for those 332 data with *I* > 2σ(*I*)]. On a final difference Fourier map, we observed no peaks higher than 0.7 e·Å^−2^. The most of the intense peaks were within 1 Å of the Si atoms. Values of thermal parameters of all atoms are within the normal interval (0.007–0.015 A^2^).

At 40.2(7) GPa, in addition to the standard data collection, we collected the diffraction when DAC was rotated around the X-ray beam direction by 90°. After the integration procedure, two reflection files were combined using program XRPEP^48^ in order to increase data completeness and redundancy. In total, we collected 2051 reflections, which were merged based upon identical indices to 900 independent reflections with *R*_int_ = 7.42%. At *d* = 0.8 Å completeness of the data was 42.1%. The amount of the collected data allowed us to refine the structure only in an isotropic approximation. The resulting ratio between data (900) and the number of parameters (97) was ~9. Six reflections were omitted from the refinement due to overlap with diamond peaks. The final structure was refined to convergence with *R*_1_ = 9.61%, *wR*_2_ = 25.99%, and GOF = 1.109 for all 900 unique reflections [*R*_1_ = 8.84%, *wR*_2_ = 24.76%, for those 789 data with *I* > 2σ(*I*)]. On a final difference Fourier map, we observed no peaks higher than 1.4 e·Å^−2^. The most of the intense peaks were within 1 Å of the Si atoms. Values of thermal parameters of all atoms are within the normal interval (0.016–0.021 A^2^).

At 44.2(4) GPa, we collected 2963 reflections in total, which were merged based upon identical indices to 2014 independent reflections with *R*_int_ = 3.95%. At *d* = 0.8 Å, completeness of the data was 37.0%. The amount of the collected data allowed us to refine the thermal parameters of silicon atoms in an anisotropic approximation, while we could refine those of oxygen atoms in an isotropic approximation. The resulting ratio between data (2014) and the number of parameters (137) was ~15. Nineteen reflections were omitted from the refinement due to overlap with diamond. The final structure was refined to convergence with *R*_1_ = 7.41%, *wR*_2_ = 13.99%, and GOF = 1.167 for all 2014 unique reflections [*R*_1_ = 5.55%, *wR*_2_ = 12.77%, for those 1562 data with *I* > 2σ(*I*)]. On a final difference Fourier map, we observed no peaks higher than 0.8 e·Å^−2^. The most of the intense peaks were within 1 Å of the Si atoms. Values of thermal parameters of all atoms are within the normal interval (0.006–0.01 A^2^).

At 49.3(8) GPa, we collected 1524 reflections in total, which were merged based upon identical indices to 968 independent reflections with *R*_int_ = 3.97%. At *d* = 0.8 Å, completeness of the data was 36.6%. The amount of the collected data allowed us to refine the thermal parameters of all atoms in an isotropic approximation. The resulting ratio between data (968) and the number of parameters (97) was ~10. Nine reflections were omitted from the refinement due to overlap with diamond. The final structure was refined to convergence with *R*_1_ = 7.59%, *wR*_2_ = 17.40%, and GOF = 1.124 for all 968 unique reflections [*R*_1_ = 6.28%, *wR*_2_ = 16.35%, for those 798 data with *I* > 2σ(*I*)]. On a final difference Fourier map, we observed no peaks higher than 0.8 e·Å^−2^. The most of the intense peaks were within 1 Å of the Si atoms. Values of thermal parameters of all atoms are within the normal interval (0.011–0.016 A^2^).

### Structure solution and refinement of coesite-V

At a single pressure point at 44.5(5) GPa, we observed the coexistence of coesite-IV and coesite-V. The orientations of the crystals were found to be very similar. Due to a small difference in the unit cell volume of coesite-IV and coesite-V, the reflections with the same *hkl*-indices belonging to two phases appeared to be close to each other.

The crystal structure of coesite-V was determined only at one pressure point at 56.7(9) GPa, where no admixture of coesite-IV was present. At higher pressures, the amount of the reflection peaks was not enough to refine the crystal structure properly, since the crystals tend to amorphize and few high-angle diffraction peaks could be observed.

In the experiment, we collected 850 reflections, which were merged based upon identical indices to 672 independent reflections with *R*_int_ = 5.08%. At *d* = 0.8 Å, completeness of the data was 30.8%. The crystal structure of coesite-IV was used as a starting model for the refinement. The crystal structure was refined against *F*^2^ on all data by full-matrix least squares with the SHELXL^47^ software. The amount of the collected data allowed us to refine the structure only in an isotropic approximation. The resulting ratio between data (672) and the number of parameters (97) was ~7. Five reflections were omitted from the refinement due to overlap with diamond. The final structure was refined to convergence with *R*_1_ = 8.92%, *wR*_2_ = 20.2%, and GOF = 1.044 for all 672 unique reflections [*R*_1_ = 7.28%, *wR*_2_ = 18.22%, for those 525 data with *I* > 2σ(*I*)]. The resulting final difference Fourier map was featureless; no peaks higher than 0.65 e·Å^−2^ were observed. Values of thermal parameters of all atoms are within the normal interval (0.018–0.023 A^2^).

### Pair-distribution functions

The pair-distribution functions were calculated using structural data (CIFs) of the corresponding crystalline phases and DiffPy software (http://www.diffpy.org/products/pdfgui.html).

### Computational details

The calculations were based on the density functional theory (DFT) and performed with the projector-augmented wave (PAW) method^49,50^, as implemented in the Vienna Ab-initio Simulation Package (VASP)^51,52^. The PAW potentials with 3*s* and 3*p* electrons of Si and 2*s* and 2*p* electrons of O treated as valence were used. The AM05 exchange-correlation functional^53^ was chosen. It provides a very good agreement of the calculated structural properties with the experimental data. We have checked, however, that the principal results of our theoretical simulations regarding the dynamical and thermodynamic stability of the studied phases of silica do not depend on the choice of the local or semi-local exchange-correlation functional: they hold in both local density approximation and the generalized gradient approximation. The Brillouin zone integration was performed on the 8 × 8 × 8 MonkhorstPack^54^ grid for stishovite/CaCl_2_-type silica and seifertite (α-PbO_2_-type) structures and on the 3 × 3 × 3 grid for coesite-IV and coesite-V. The plane-wave energy cutoff was set to 600 eV, i.e., by 50% higher than the default VASP value. The energies of the fully relaxed structures were used together with their volumes and pressures to calculate the enthalpies via the standard definition. Stishovite is considered as the ground-state structure of SiO_2_ at a pressure below 35 GPa. Assuming the typical accuracy of ab initio calculations of the lattice parameters of ~0.01 Å, our simulations show that it spontaneously undergoes a tetragonal transformation into a CaCl_2_-type structure between 35 and 40 GPa, in agreement with earlier theoretical work. All enthalpies were calculated relative to the enthalpy of the ground-state structure at the corresponding pressure (Supplementary Fig. 5). The calculated energies, pressures, and volumes were also used to fit theoretical data using the third-order Birch–Murnaghan equation of state.

Phonon dispersion relations of coesite-IV and coesite-V phases were calculated at 0 K in the harmonic approximation, using the small displacement method, as implemented in Phonopy code^55^. Crystal structures of the phases were first fully relaxed with constrained unit cell volumes corresponding to the experimental volumes at the respective pressure. Then a force field was obtained in a 2 × 2 × 2 supercell (384 atoms), based on 144 single atomic displacements with an amplitude of 0.01 Å. As our main task was to investigate the dynamic stability/instability of the new silica phases at room temperature, we did not take into account the LO–TO splitting in high-frequency optical branches. Reciprocal space was sampled using the 2 × 2 × 2 Γ-centered *k*-point mesh.

Bonding was characterized by the Bader analysis^56^, which defines that the electronic charge belongs to the particular atom, if it is encountered inside the so-called Bader volumes enclosed by the zero-flux surfaces perpendicular to the minima of the charge density. The so-obtained Bader charge is considered as a good approximation for the total all-electron charge of the corresponding atom.

### Computational results

The calculated crystal structure parameters of coesite-IV and coesite-V phases of SiO_2_ are presented in Supplementary Table 10. Very good agreement between theoretical and experimental data confirms the reliability of the adopted theoretical approximations for the simulation presented in this work.

Vibrational spectra of coesite-IV at pressure *P* = 39 GPa and coesite-V at *P* = 57 GPa are shown in Supplementary Fig. 10a, b, respectively. No imaginary frequencies were observed in either of the phases, i.e., both of them are dynamically stable at pressures where they are observed experimentally. However, our results suggest that coesite-IV and coesite-V are metastable phases of silica. Supplementary Fig. 5 shows the differences between the enthalpies of SiO_2_ phases considered in this study, coesite-IV, coesite-V, as well as seifertite (α-PbO_2_-type structure) relative to the enthalpy of stishovite/CaCl_2_-type silica. One can see that the former two are much higher in enthalpy in comparison to the latter. At the same time, the stability of the coesite-IV phase relative to the coesite-V phase is correctly reproduced in our calculations. Indeed, the former is more stable at lower pressure, while the latter has a lower enthalpy above 38 GPa. Note that the optimization (relaxation) of the crystal structure of coesite-IV at unit cell volume of 352.35 Å^3^ resulted in its spontaneous transformation into coesite-V phase. At this volume (corresponding to experimental pressure of 49 GPa, i.e., the highest experimental pressure at which coesite-IV phase is observed experimentally) as well as at lower volumes (higher pressures), coesite-IV phase exhibits instability resulting in coesite-IV> coesite-V transition. The direct pathway between the phases is shown in Supplementary Movie 1.

The calculated EOSs of coesite-IV and coesite-V phases of SiO_2_ (Supplementary Fig. 6) show good agreement with the experiment. Calculated bulk moduli and their pressure derivatives are summarized in Supplementary Table 11. In particular, our theoretical results predict that coesite-IV and coesite-V phases of SiO_2_ are much softer than stishovite/CaCl_2_-type silica and seifertite.

Analyzing the bonding of the investigated silica phases by the Bader analysis (Supplementary Fig. 10), we conclude that the ionicity of the coesite-IV and coesite-V phases does not change qualitatively, as compared with that of stishovite/CaCl_2_ and α-PbO_2_ phases.

## Electronic supplementary material

Supplementary Information

Description of Additional Supplementary Files

Supplementary Movie 1

Supplementary Data 1

Supplementary Data 2

Supplementary Data 3

Supplementary Data 4

Supplementary Data 5

Supplementary Data 6

Supplementary Data 7

Supplementary Data 8

Supplementary Data 9

Supplementary Data 10

Supplementary Data 11

Supplementary Data 12

## Data Availability

The X-ray crystallographic coordinates for structures reported in this article have been deposited at the Inorganic Crystal Structure Database (ICSD) under deposition number CSD (1860556–1860561). These data can be obtained from CCDC’s and FIZ Karlsruhe’s free service for viewing and retrieving structures (https://www.ccdc.cam.ac.uk/structures/). The crystallographic information (CIF-files and the corresponding CheckCIF reports) is also available as Supplementary Data 1–12.
