# Supplementary material for: Metastable silica high pressure polymorphs as structural proxies of deep Earth silicate melts
Source: Nat Commun. 2018 Nov 15;9:4789. doi: 10.1038/s41467-018-07265-z (PMC6237875; doi:10.1038/s41467-018-07265-z)
Supplement: Supplementary file 1 — Supplementary Information [file 41467_2018_7265_MOESM1_ESM.pdf]

Metastable silica high pressure polymorphs as structural proxies of deep Earth silicate melts

E. Bykova *et al.*

Supplementary information

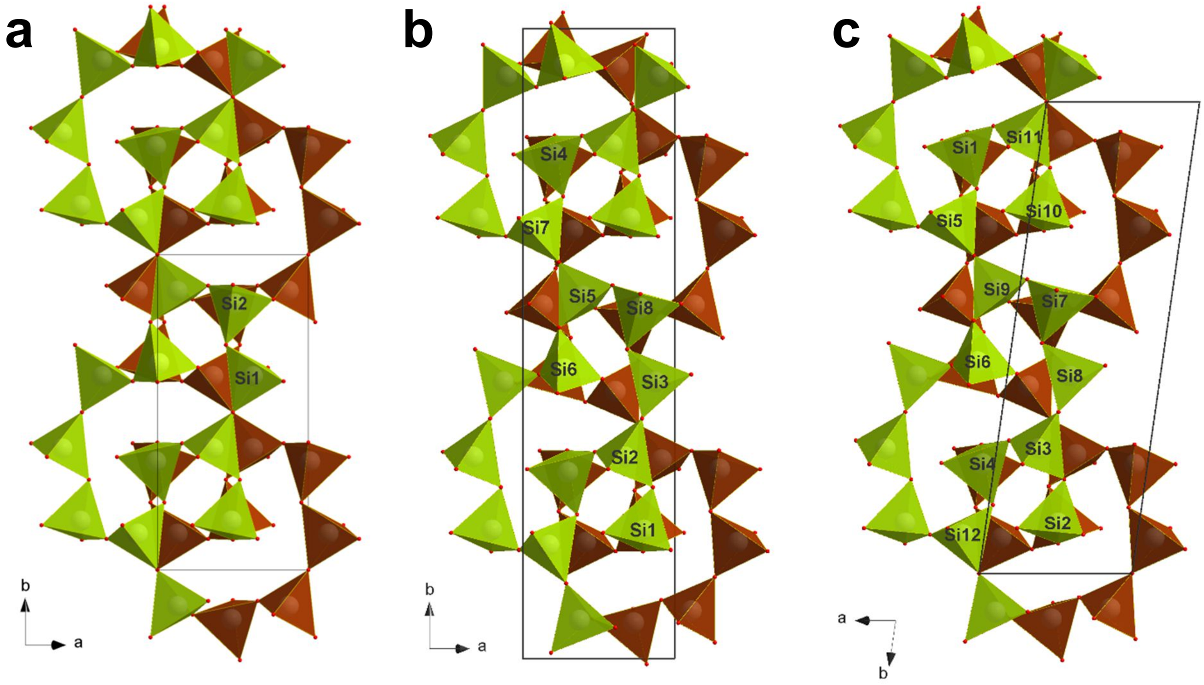

Supplementary Figure 1. Crystal structures of coesite-I (a, 20.3(15) GPa)[1], coesite-II (b, 27.52(13) GPa)[1], and coesite-III (c, 27.9(5) GPa). Although structural elements in all three phases are very similar, there are significant differences: in coesite-I two crystallographically independent silicon atoms locate in relatively weakly distorted tetrahedra (bond angle variance, BAV, equals 2.0 and 12.1 squared degrees), in coesite –II there are eight different significantly distorted tetrahedra (BAV ranges from 6.3 to 43.0 squared degrees), and in triclinic coesite-III there are twelve types of SiO_4 polyhedra distorted stronger (BAV ranges from 10.0 to 62.5 squared degrees). Bond angle variance was determined with VESTA software[2]. The value is calculated according to the following formula:

$$\sigma^2 = \frac{1}{m-1}\sum_{i=1}^{m}(\phi_i - \phi_0)^2$$

where m is (number of faces in the polyhedron)*3/2 (i.e., number of bond angles), ϕ_i is the ith bond angle, and ϕ_0 is the ideal bond angle for a regular polyhedron[3]

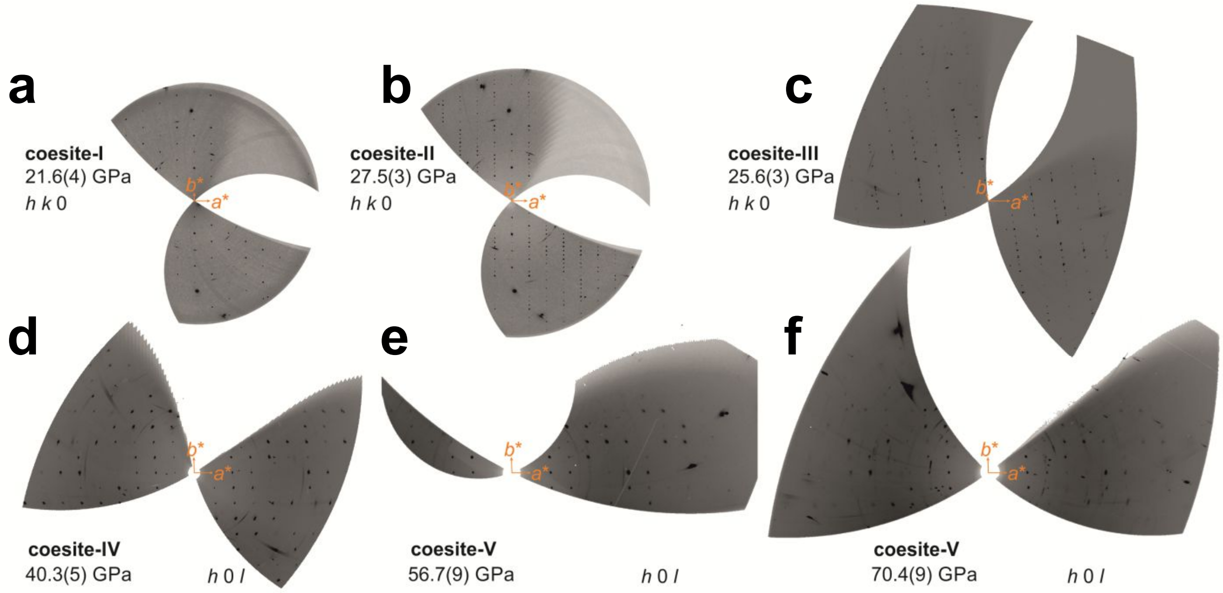

Supplementary Figure 2. Reciprocal space reconstructions for coesite phases. For coesite-I, II, and III having similar crystal structures we selected *h k* 0 plane for reconstructions (**a**, **b**, **c**, respectively). Above 22 GPa additional reflections with ½ ***b**** appear (**b**), manifesting the transition from coesite-I to coesite-II with doubling of unit cell parameter *b*. One can see emerging of the new reflections above 25 GPa along the ***b****-axis belonging to coesite-III (**c**). For coesite-IV (**d**) and V (**e** and **f**) we selected *h* 0 *l* plane in order to keep approximately the orientation of the initial crystal. Upon compression the quality of the coesite-V crystal decreases, however it doesn't amorphize up to the highest pressure reached (70.4(9) GPa).

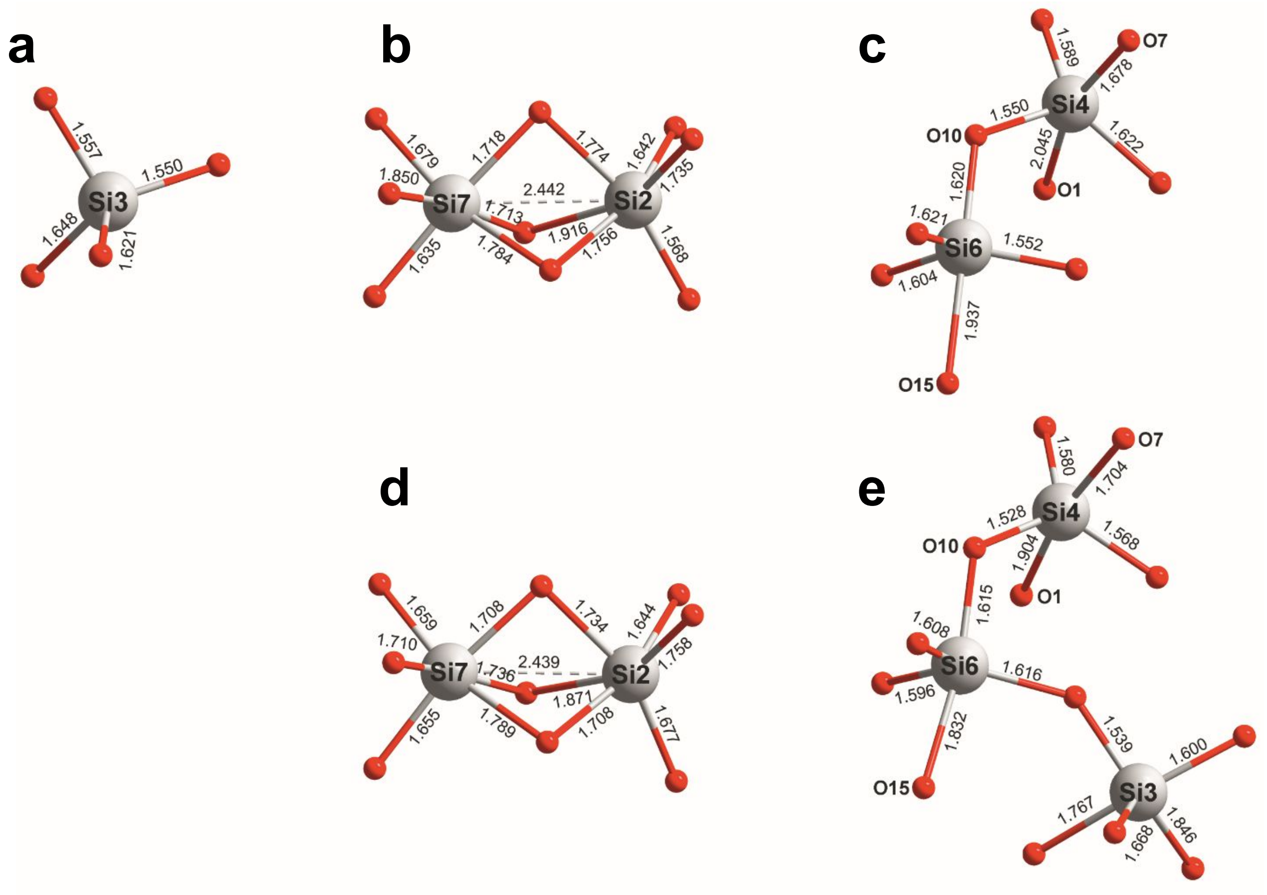

Supplementary Figure 3. Ball and stick models of polyhedra representing building blocks of the structures of coesite-IV and coesite-V. Coesite-IV (dimensions as at 44.2(4) GPa): SiO_4 tetrahedron (**a**); two SiO_6 octahedra sharing a face (**b**); two SiO_5 polyhedra sharing a corner (**c**); coesite-V (dimensions as at 56.8(9) GPa): two SiO_6 octahedra sharing a face (**d**); three SiO_5 polyhedra with common corners (**e**).

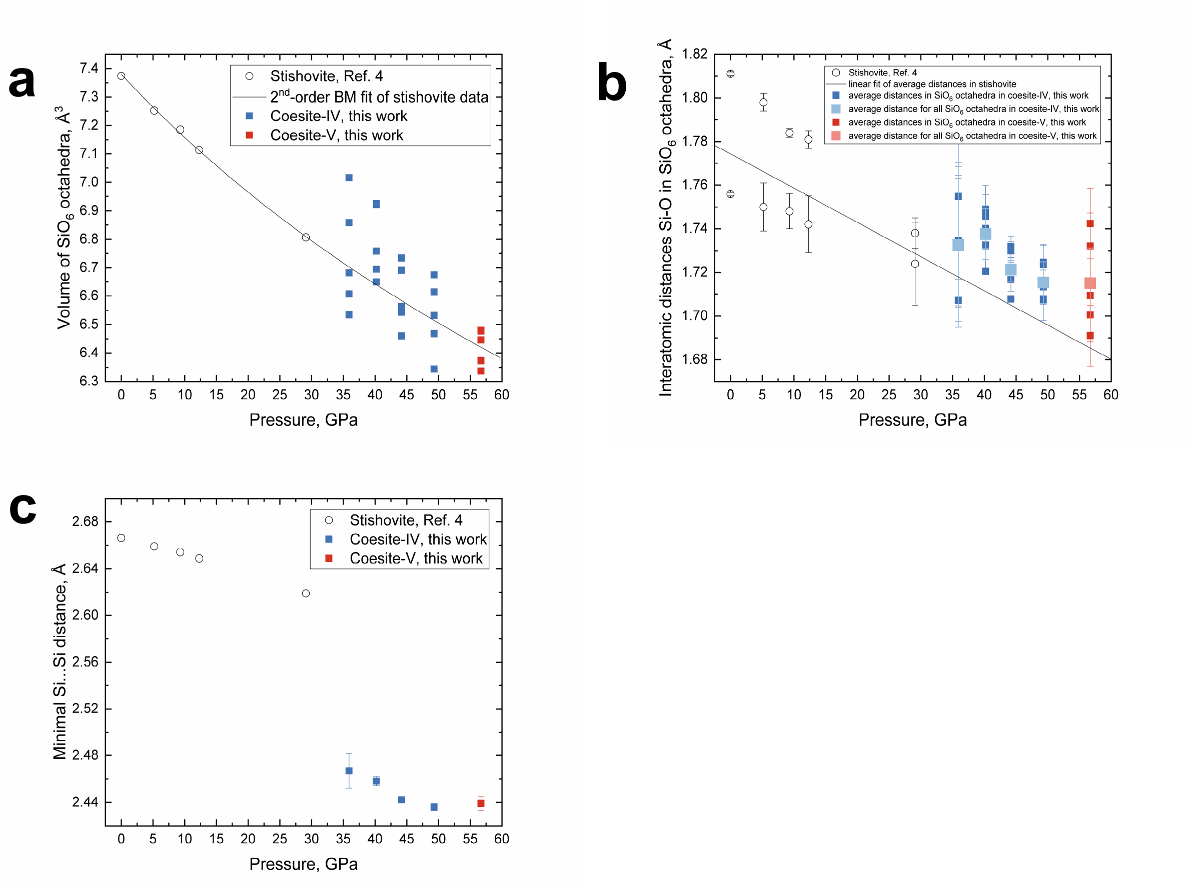

Supplementary Figure 4. Geometric characteristics of crystal structures of coesite-IV (blue squares) and coesite-V (red squares) compared to stishovite (open circles). a, Scatter graph shows volumes of SiO_6 octahedra and black line represents the fit of volumes in stishovite with the 2rd order Birch-Murnaghan equation of state (V_0= 7.38(1) $Å^3$, $K_{0,300}$=308(3) GPa)[4]. **b,** Scatter graph represents individual Si-O distances in SiO_6 octahedra. For coesite phases the distances for each octahedron were averaged and only these average distances are shown. Light blue and rose squares are the Si-O distances averaged for all SiO_6 octahedra in coesite high-pressure phases. Black line is the linear extrapolation of the average Si-O distance in stishovite[4]. **c,** Scatter graph shows minimal Si…Si distances.

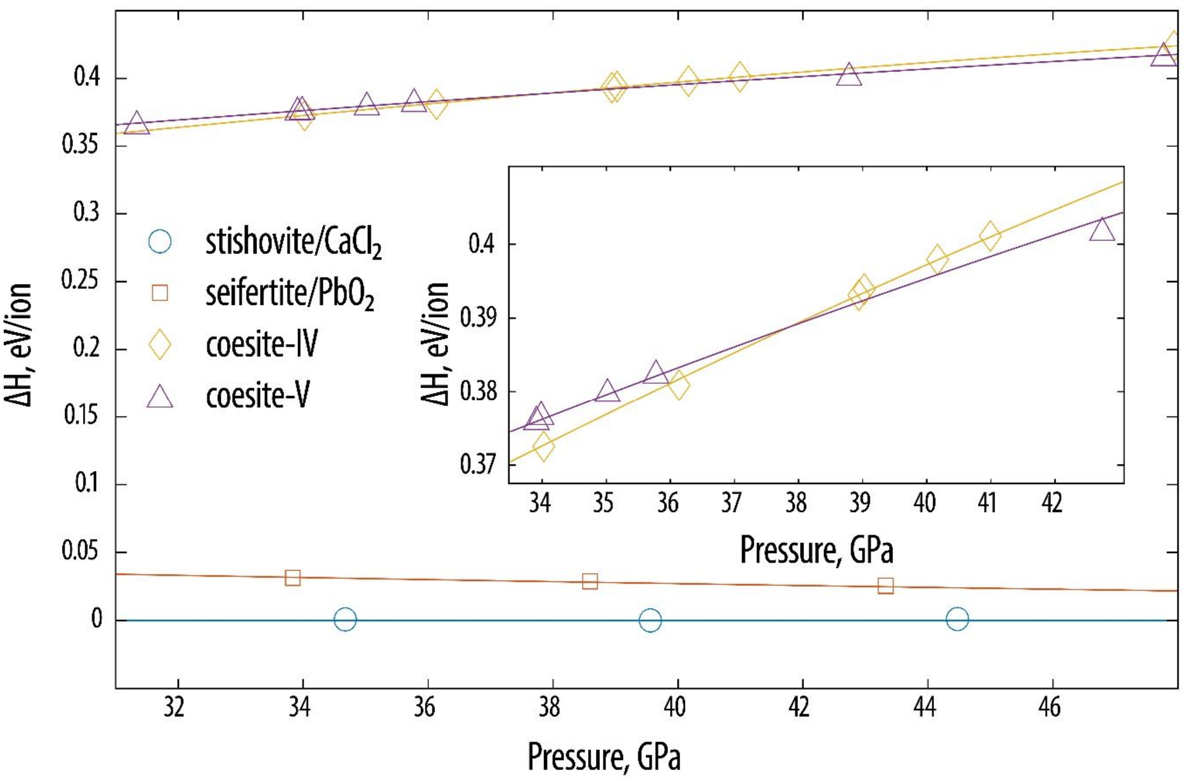

Supplementary Figure 5. Enthalpy difference between different silica phases – stishovite/$CaCl_2$-type, seifertite (α-PbO_2-type), coesite-IV, and coesite-V. The data points are the DFT results calculated with the AM05 functional.

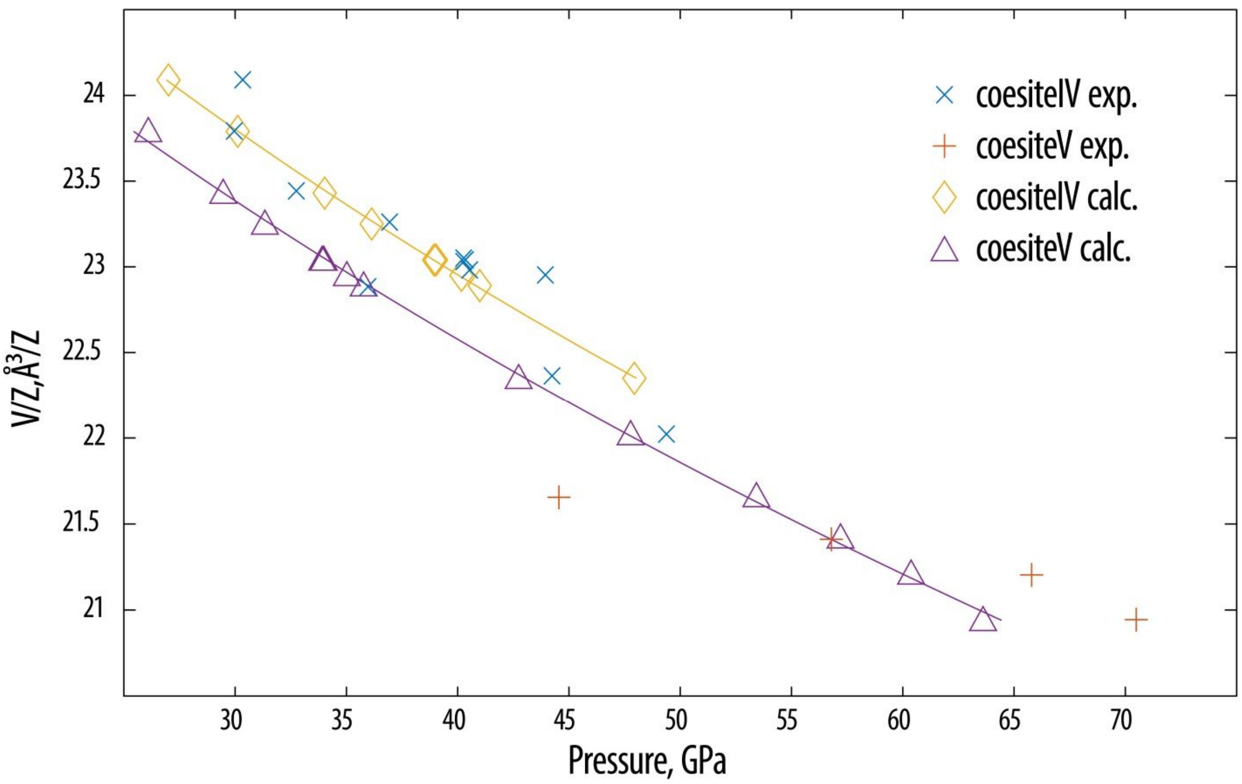

Supplementary Figure 6. Pressure dependence of volumes (per formula unit) of coesite-IV and coesite-V as calculated ab initio (at 0 K). Calculated values shown by yellow diamonds (coesite-IV) and purple triangles (coesite-V). Experimental values shown by crosses.

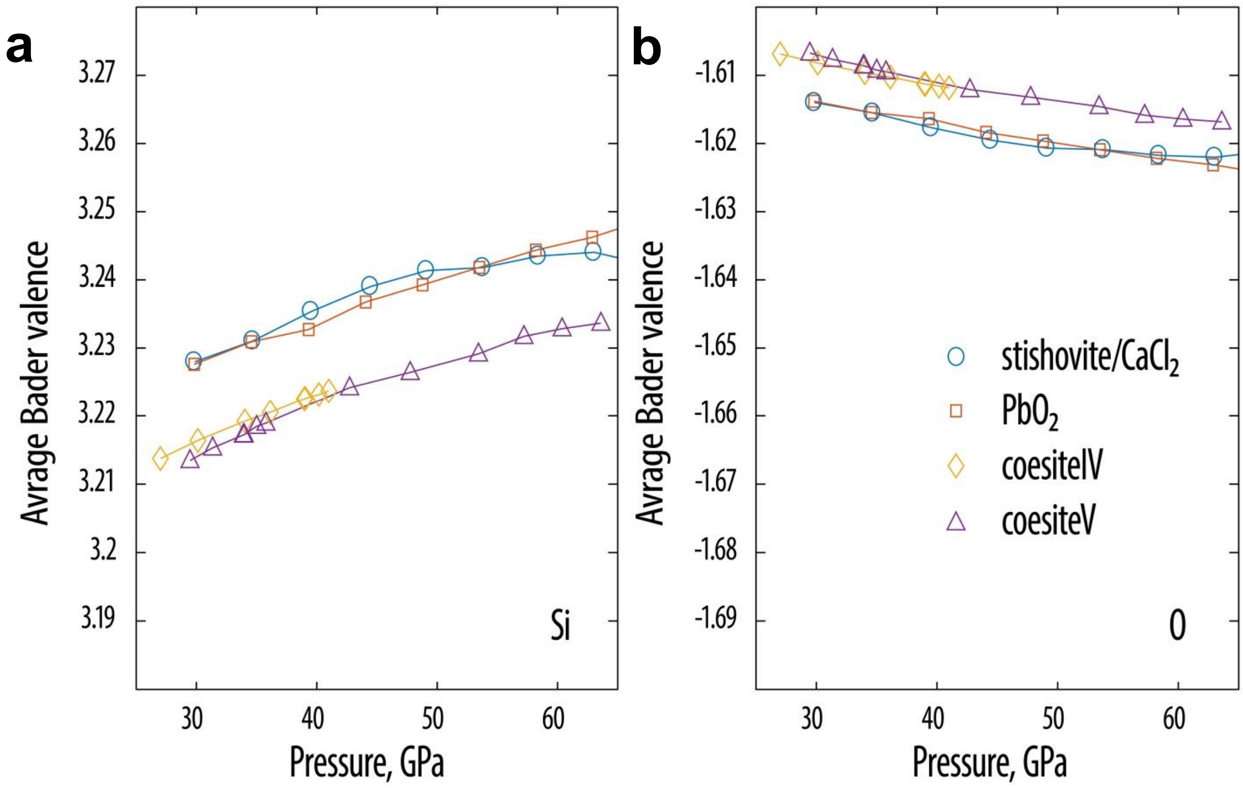

Supplementary Figure 7. Charges of silicon (**a**) and oxygen (**b**) atoms in different silica polymorphs as function of pressure as obtained by Bader analysis based on *ab initio* calculations.

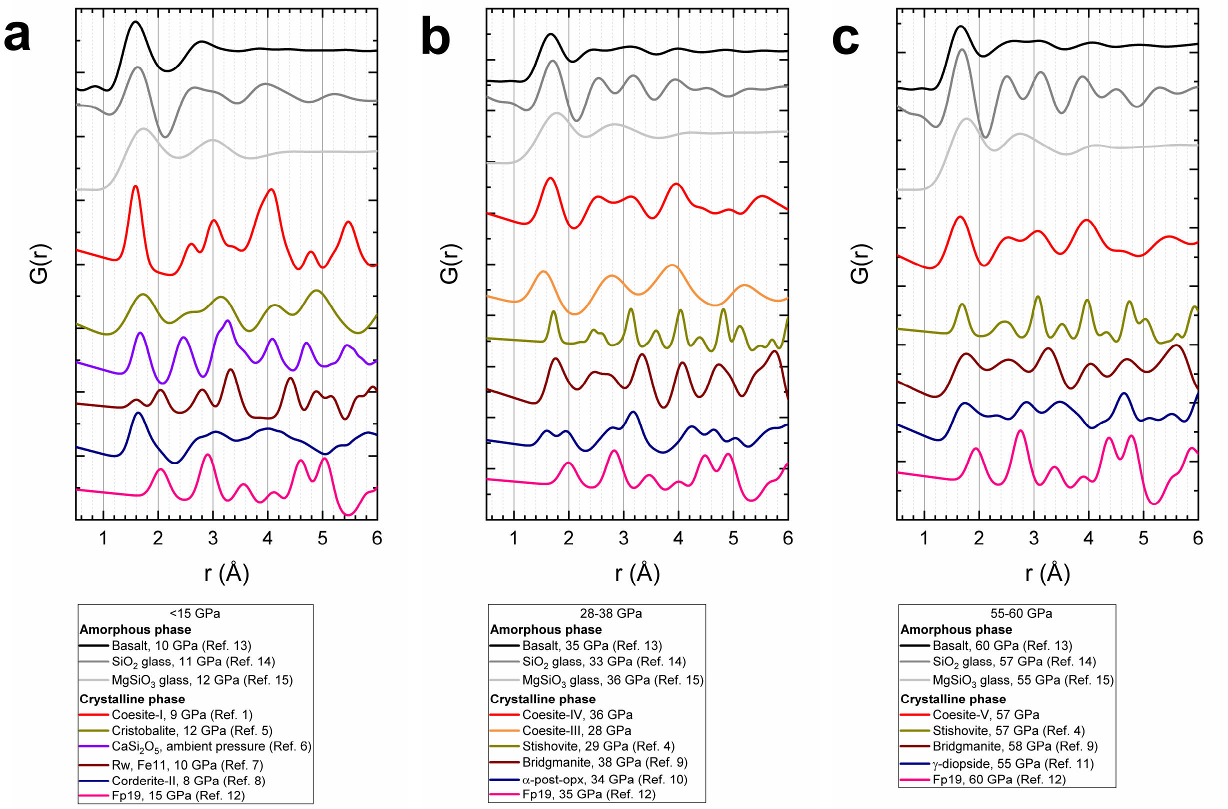

Supplementary Figure 8. Pair distribution functions calculated for silica polymorphs (this work and Refs. 1,4,5**), silicates**[6–11] **and ferropericlase (Fp19)**[12] **compared with those for basalt**[13]**, silica**[14] **and $MgSiO_3$**[15] **glass measured at different pressures: a**, below 15 GPa; **b**, in the range from 28 to 38 GPa; **c**, in the range from 55 to 60 GPa.

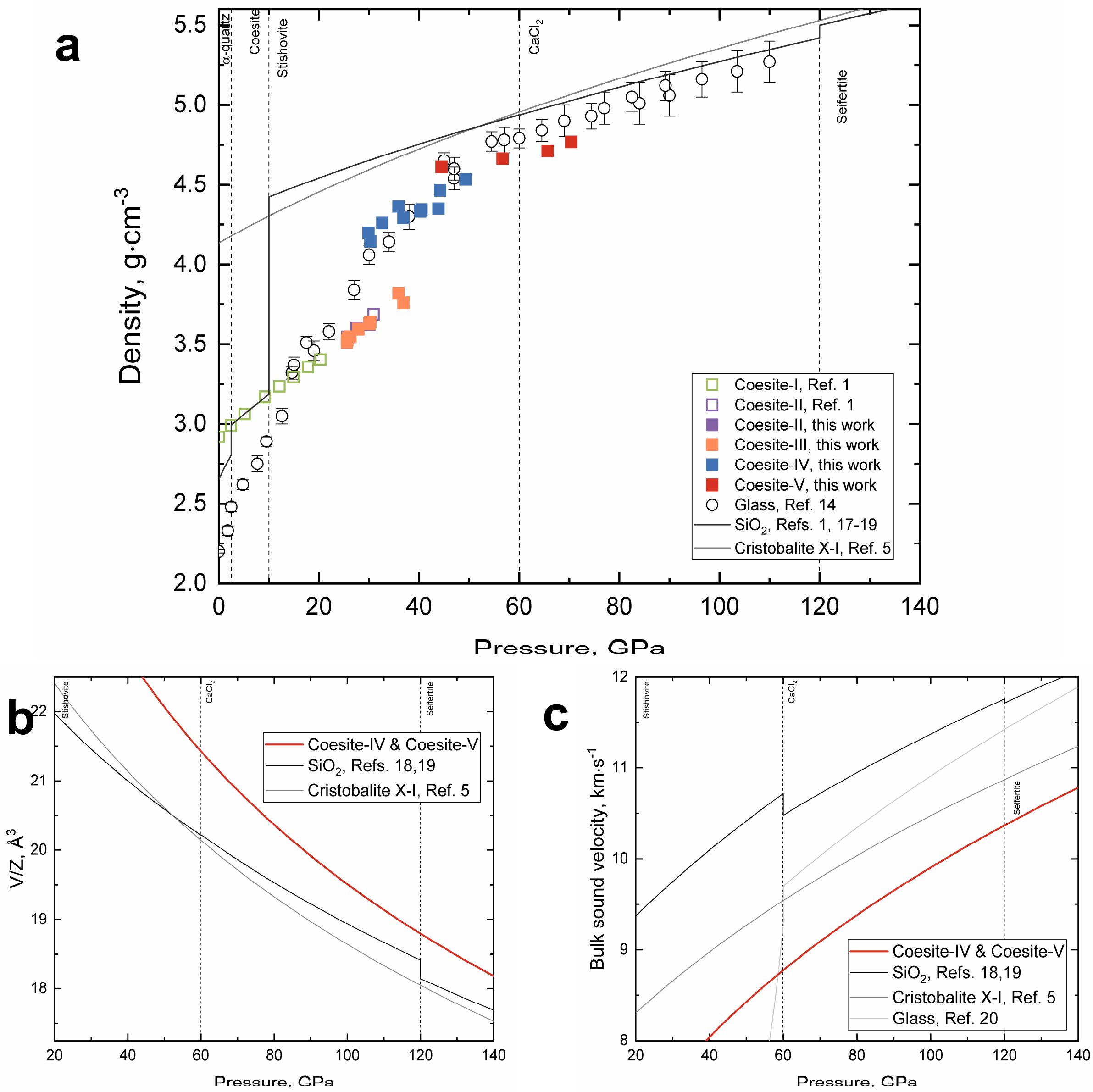

Supplementary Figure 9. Elasticity of various silica phases compared to high-pressure phases of coesite. a, Scatter graph represent experimental density values taken for coesite phases (open squares are previous data reported in Refs. 1,16 and filled squares are the data collected in this work) and silica glass (open circles)[14]. Black line shows density of SiO_2 equilibrium phases[1,17–19]. Grey line represents calculated density of cristobalite X-I[5]. **b**, Solid black line shows pressure dependence of volumes (per formula unit) calculated using experimental equations of state of equilibrium silica phases (stishovite, $CaCl_2$-type, and seifertite)[19] and dark grey line is the similar dependence for cristobalite X-I[5]. Combined pressure-volume data for coesite-IV and coesite-V was fitted with 2nd order Birch-Murnaghan equation of state ($V_{32.7}/Z$=23.44(3) Å^3, $K_{32.7}$=254(9) GPa) shown on the graph by solid red line. **c**, Solid lines show pressure dependence of bulk sound velocities calculated for equilibrium silica phases (stishovite, $CaCl_2$-type, and seifertite; black line)[19], silica glass (dark grey line)[20], cristobalite X-I (light grey line)[5] and for combined dataset coesite-IV + coesite-V.

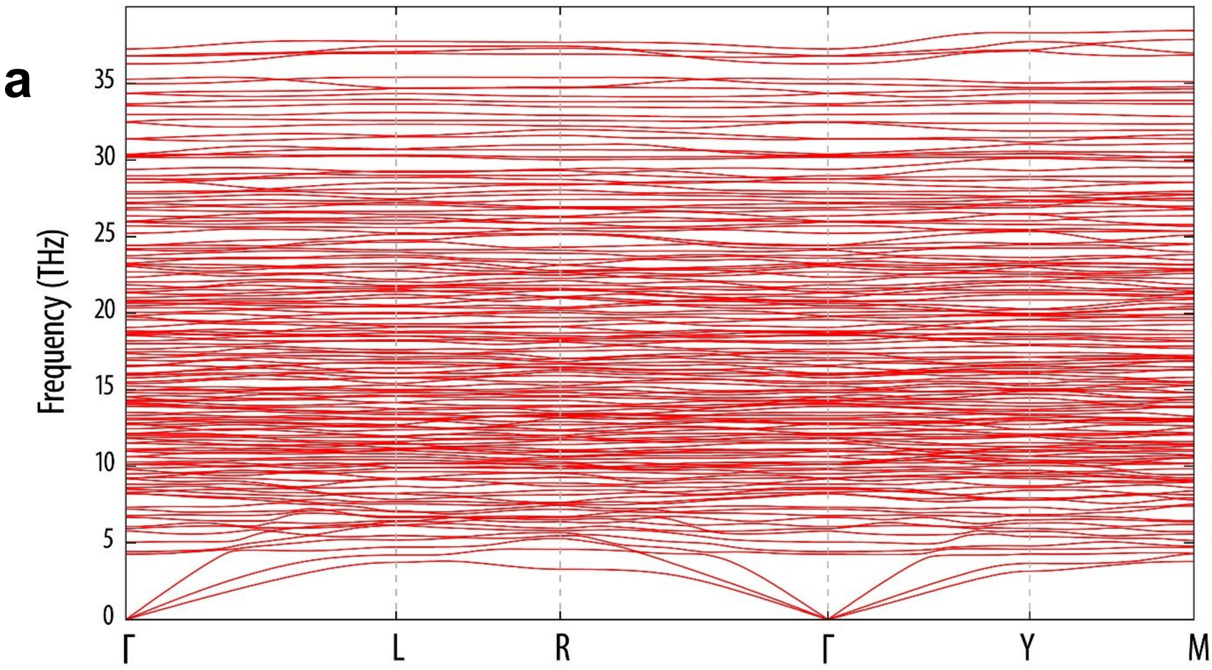

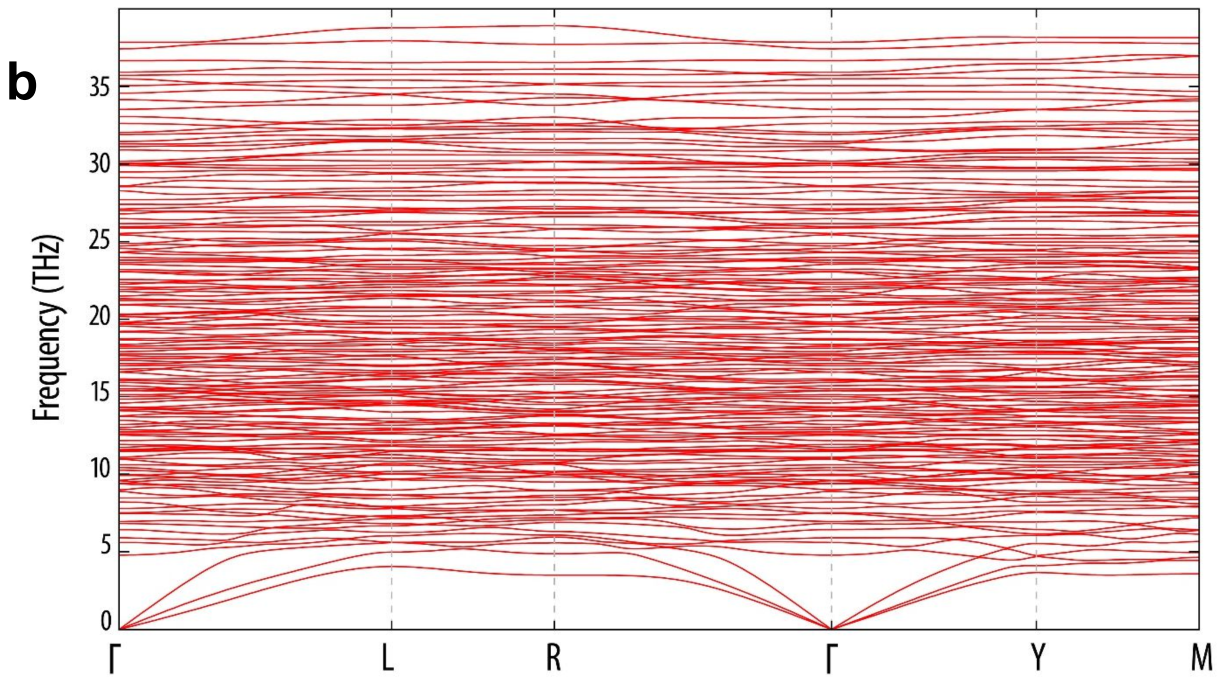

Supplementary Figure 10. Phonon dispersion relation calculated in the harmonic approximation for coesite-IV (**a**) and coesite-V (**b**). The unit cell volume of coesite-IV is 368.50 Å^3 and corresponds to a theoretical pressure of 39 GPa at 0 K; the unit cell volume of coesite-V is 342.57 Å^3 and corresponds to a theoretical pressure of 57 GPa at 0 K (see also Supplementary Table 4).

Supplementary Table 1. Summary of the high-pressure single-crystal XRD experiments on coesite

Crystal #	Pressure, GPa	Phase composition	Experimental details<br>(beamline, wavelength, detector, focusing, beamsize)	Data collection<br>(ω range, step size)
**DAC#1**				
c1	5.8(5)	coe-I	P02.2, λ = 0.29004 Å, MAR345dtb, 2.5(H)x1.4(V) μm^2	from -26 to +26°, 1° step
c1	27.9(5)	coe-III	P02.2, λ = 0.29004 Å, MAR345dtb, 2.5(H)x1.4(V) μm^2	from -26 to +26°, 1° step
c1	35.9(7)	coe-III<br>coe-IV	P02.2, λ = 0.29004 Å, MAR345dtb, 2.5(H)x1.4(V) μm^2	from -26 to +26°, 1° step
c1	44.5(5)	coe-IV<br>coe-V	P02.2, λ = 0.29004 Å, MAR345dtb, 2.5(H)x1.4(V) μm^2	from -26 to +26°, 1° step
c1	57.1(6)	coe-V	P02.2, λ = 0.29004 Å, MAR345dtb, 2.5(H)x1.4(V) μm^2	from -26 to +26°, 1° step
**DAC#2**				
c1	14.2(3)	coe-I	P02.2, λ = 0.29056 Å, MAR345dtb, 2.6(H)x1.3(V) μm^2	from -40 to +40°, 1° step
c1	21.6(4)	coe-I	P02.2, λ = 0.29056 Å, MAR345dtb, 2.6(H)x1.3(V) μm^2	from -40 to +40°, 1° step
c1	27.5(3)	coe-II	P02.2, λ = 0.29056 Å, MAR345dtb, 2.6(H)x1.3(V) μm^2	from -40 to +40°, 1° step
c2	27.9(4)	coe-II<br>coe-III	P02.2, λ = 0.29004 Å, MAR345dtb, 2.5(H)x1.4(V) μm^2	from -40 to +40°, 1° step
c1	29.9(6)	coe-II<br>coe-IV	P02.2, λ = 0.29004 Å, MAR345dtb, 2.5(H)x1.4(V) μm^2	from -40 to +40°, 1° step
c1	32.7(5)	coe-II<br>coe-IV	P02.2, λ = 0.29004 Å, MAR345dtb, 2.5(H)x1.4(V) μm^2	from -40 to +40°, 1° step
c1	43.8(5)	coe-IV	P02.2, λ = 0.29056 Å, MAR345dtb, 2.6(H)x1.3(V) μm^2	from -40 to +40°, 1° step
c1	43.9(4)	coe-IV	P02.2, λ = 0.29056 Å, MAR345dtb, 2.6(H)x1.3(V) μm^2	from -40 to +40°, 1° step
**DAC#3**				
c1	5.3(5)	coe-I	ID09A, λ = 0.41273 Å, MAR555, 10(H)x10(V) μm^2	from -40 to +40°, 1° step
c2	5.5(5)	coe-I	ID09A, λ = 0.41273 Å, MAR555, 10(H)x10(V) μm^2	from -40 to +40°, 1° step
c1	25.6(3)	coe-II<br>coe-III	ID09A, λ = 0.41273 Å, MAR555, 10(H)x10(V) μm^2	from -40 to +40°, 1° step
c1	25.6(3)	coe-III	ID09A, λ = 0.41273 Å, MAR555, 10(H)x10(V) μm^2	from -40 to +40°, 1° step,<br>10x less intensity of the primary beam
c1	25.7(3)	coe-II<br>coe-III	ID09A, λ = 0.41273 Å, MAR555, 10(H)x10(V) μm^2	from -40 to +40°, 1° step,<br>DAC rotated by 90°
c1	25.8(3)	coe-III	ID09A, λ = 0.41273 Å, MAR555, 10(H)x10(V) μm^2	from -40 to +40°, 1° step,<br>10x less intensity of the primary beam,<br>DAC rotated by 90°
c2	26.3(3)	coe-II<br>coe-III	ID09A, λ = 0.41273 Å, MAR555, 10(H)x10(V) μm^2	from -40 to +40°, 1° step
c1	30.3(4)	coe-III<br>coe-IV	ID09A, λ = 0.41273 Å, MAR555, 10(H)x10(V) μm^2	from -40 to +40°, 1° step
c2	30.1(4)	coe-II<br>coe-III	ID09A, λ = 0.41273 Å, MAR555, 10(H)x10(V) μm^2	from -40 to +40°, 1° step
c1	36.9(4)	coe-III<br>coe-IV	ID09A, λ = 0.41273 Å, MAR555, 10(H)x10(V) μm^2	from -40 to +40°, 1° step
c1	40.3(5)	coe-IV	ID09A, λ = 0.41273 Å, MAR555, 10(H)x10(V) μm^2	from -40 to +40°, 1° step
c1	40.5(4)	coe-IV	ID09A, λ = 0.41273 Å, MAR555, 10(H)x10(V) μm^2	from -40 to +40°, 1° step,<br>10x less intensity of the primary beam,
c1	40.2(7)	coe-IV	ID09A, λ = 0.41273 Å, MAR555, 10(H)x10(V) μm^2	from -40 to +40°, 1° step,<br>DAC rotated by 90°
c1	40.2(7)	coe-IV	ID09A, λ = 0.41273 Å, MAR555, 10(H)x10(V) μm^2	from -40 to +40°, 1° step,<br>10x less intensity of the primary beam,<br>DAC rotated by 90°
c1	44.2(4)	coe-IV	P02.2, λ = 0.28965 Å, Perkin Elmer XRD1621, 7.5(H)x3.5(V) μm^2	from -40 to +29°, 0.25° step
c1	49.3(8)	coe-IV	P02.2, λ = 0.28965 Å, Perkin Elmer XRD1621, 7.5(H)x3.5(V) μm^2	from -40 to +29°, 0.25° step
c1	56.7(9)	coe-V	P02.2, λ = 0.28965 Å, Perkin Elmer XRD1621, 7.5(H)x3.5(V) μm^2	from -40 to +29°, 0.25° step
c1	65.7(9)	coe-V	P02.2, λ = 0.28965 Å, Perkin Elmer XRD1621, 7.5(H)x3.5(V) μm^2	from -40 to +29°, 0.25° step
c1	70.4(9)	coe-V	P02.2, λ = 0.28965 Å, Perkin Elmer XRD1621, 7.5(H)x3.5(V) μm^2	from -40 to +29°, 0.5° step

Supplementary Table 2. Unit cell parameters of high-pressure phases of coesite summarized from current experimental results

Pressure, GPa	*a*, Å	*b*, Å	*c*, Å	α, °	β, °	γ, °	*V*, $Å^3$	*V*/*Z*, $Å^3$	Sample ID
**Coesite-II, *P*2$_1$/*n*, *Z* = 32**									
25.6(3)	6.632(3)	23.401(5)	6.891(6)	90	121.67(9)	90	910.2(9)	28.44(3)	DAC#3, c1
25.7(3)	6.613(4)	23.352(6)	6.837(3)	90	121.44(8)	90	900.9(8)	28.15(3)	DAC#3, c1
26.3(3)	6.566(1)	23.320(2)	6.848(2)	90	120.75(3)	90	901.1(3)	28.159(9)	DAC#3, c1
30.1(4)	6.534(2)	23.157(3)	6.783(4)	90	120.78(6)	90	881.7(6)	27.55(2)	DAC#3, c2
**Coesite-III, *P*-1, *Z* = 24**									
25.6(3)	6.591(3)	17.879(4)	6.881(4)	82.63(3)	121.11(5)	87.12(3)	682.0(5)	28.42(2)	DAC#3, c1
25.6(3)	6.587(2)	17.881(3)	6.853(3)	82.56(2)	120.96(4)	87.09(2)	679.8(3)	28.32(1)	DAC#3, c1
25.8(3)	6.583(3)	17.894(4)	6.804(3)	82.46(3)	120.70(5)	86.98(2)	676.5(4)	28.19(2)	DAC#3, c1
25.7(3)	6.604(2)	17.901(3)	6.825(2)	82.65(2)	120.90(3)	86.84(2)	679.7(3)	28.32(1)	DAC#3, c1
26.3(3)	6.5741(7)	17.848(4)	6.815(3)	82.62(3)	120.68(3)	86.95(2)	675.3(4)	28.14(2)	DAC#3, c2
27.9(5)	6.571(3)	17.790(7)	6.766(4)	82.14(4)	120.81(6)	87.13(3)	666.2(6)	27.76(3)	DAC#1, c1
27.9(4)	6.551(1)	17.764(4)	6.789(2)	82.57(2)	120.79(3)	86.97(2)	666.5(3)	27.77(1)	DAC#2, c2
30.3(4)	6.564(2)	17.640(5)	6.710(3)	82.11(3)	119.99(4)	86.36(3)	658.5(4)	27.44(2)	DAC#3, c1
30.1(4)	6.537(2)	17.754(7)	6.755(4)	82.71(4)	120.81(5)	86.53(3)	660.5(5)	27.52(2)	DAC#3, c2
35.9(7)	6.496(4)	17.237(9)	6.621(5)	82.69(5)	120.38(7)	86.52(4)	627.6(7)	26.15(3)	DAC#1, c1
36.9(4)	6.466(5)	17.456(7)	6.666(5)	83.16(5)	120.40(8)	86.17(5)	637.3(7)	26.55(3)	DAC#3, c1
**Coesite-IV, *P*-1, *Z* = 16**									
29.9(6)	6.741(4)	7.045(4)	8.693(4)	69.57(4)	82.22(4)	80.96(5)	380.6(3)	23.79(2)	DAC#2 c1
30.3(4)	6.714(2)	7.068(3)	8.757(4)	70.06(4)	83.17(3)	81.83(3)	385.5(2)	24.09(1)	DAC#3, c1
32.7(5)	6.688(4)	7.004(4)	8.653(4)	69.82(5)	82.58(4)	81.60(5)	375.0(4)	23.44(3)	DAC#2 c1
35.9(7)	6.524(9)	6.942(9)	8.674(5)	70.07(8)	84.05(7)	83.6(1)	366.1(7)	22.88(4)	DAC#1, c1
36.9(4)	6.640(1)	6.996(3)	8.665(3)	69.51(3)	83.12(2)	81.94(2)	372.2(2)	23.26(1)	DAC#3, c1
40.3(5)	6.596(1)	6.996(3)	8.660(3)	69.17(4)	83.20(2)	81.76(2)	368.6(2)	23.04(1)	DAC#3, c1
40.5(4)	6.598(1)	6.982(3)	8.647(4)	69.27(4)	83.16(3)	81.75(2)	367.7(3)	22.98(2)	DAC#3, c1
40.2(7)	6.6017(9)	7.005(2)	8.649(3)	69.09(3)	83.13(2)	81.75(2)	368.8(2)	23.05(1)	DAC#3, c1
40.2(7)	6.5989(8)	7.000(2)	8.648(3)	69.14(3)	83.15(2)	81.80(2)	368.5(2)	23.03(1)	DAC#3, c1
43.9(4)	6.496(6)	6.951(6)	8.563(6)	74.03(7)	83.99(6)	81.90(7)	367.1(5)	22.95(3)	DAC#2 c1
44.2(4)	6.5586(4)	6.9029(7)	8.5429(8)	69.599(9)	83.035(6)	81.933(6)	357.83(5)	22.364(3)	DAC#3, c1
49.3(8)	6.5175(8)	6.875(1)	8.494(2)	69.61(2)	83.29(1)	81.99(1)	352.35(9)	22.022(6)	DAC#3, c1
**Coesite-V, *P*-1, *Z* = 16**									
44.5(5)	6.34(1)	6.71(1)	8.610(6)	72.21(9)	85.47(8)	83.3(1)	346.4(8)	21.65(5)	DAC#1, c1
56.7(9)	6.403(2)	6.768(2)	8.400(3)	72.40(3)	84.02(3)	81.64(3)	342.6(2)	21.41(1)	DAC#3, c1
65.7(9)	6.397(7)	6.754(7)	8.34(1)	72.8(1)	83.8(1)	81.3(1)	339.2(7)	21.20(4)	DAC#3, c1
70.4(9)	6.366(4)	6.785(8)	8.281(9)	71.9(1)	83.5(1)	81.0(1)	335.0(6)	20.94(4)	DAC#3, c1

Supplementary Table 3. Details of crystal structure refinements of coesite high-pressure phases

	**coesite-III**	**coesite-IV**				**coesite-V**
**Pressure, GPa**	27.9(5)	35.9(7)	40.2(7)	44.2(4)	49.3(8)	56.7(9)
**Crystal system**	triclinic	triclinic	triclinic	triclinic	triclinic	triclinic
**Space group**	*P*-1	*P*-1	*P*-1	*P*-1	*P*-1	*P*-1
a **(Å)**	6.571(3)	6.524(9)	6.5989(8)	6.5586(4)	6.5175(8)	6.4034(18)
b **(Å)**	17.790(7)	6.942(9)	7.000(2)	6.9029(7)	6.8754(12)	6.768(2)
c **(Å)**	6.766(4)	8.674(5)	8.649(3)	8.5429(8)	8.4943(15)	8.399(3)
**α (°)**	82.14(4)	70.07(8)	69.14(3)	69.599(9)	69.612(16)	72.40(3)
**β (°)**	120.81(6)	84.05(7)	83.153(16)	83.035(6)	83.289(12)	84.02(3)
**γ (°)**	87.13(3)	83.63(11)	81.801(16)	81.933(6)	81.990(12)	81.64(3)
V **(Å^3)**	666.2(6)	366.1(8)	368.46(17)	357.83(6)	352.35(10)	342.6(2)
Z	24	16	16	16	16	16
***F*(000)**	720	480	480	480	480	480
**Theta range for data collection (°)**	2.839 to 11.146	2.902 to 11.146	1.816 to 19.601	1.910 to 19.569	1.919 to 12.872	1.883 to 11.939
**Completeness to d = 0.8 Å, %**	0.253	0.255	0.421	0.37	0.366	0.308
**Index ranges**	$-8 < h < 8$,	$-7 < h < 5$,	$-9 < h < 10$,	$-13 < h < 14$,	$-9 < h < 10$,	$-8 < h < 9$,
	$-23 < k < 23$,	$-7 < k < 6$,	$-6 < k < 8$,	$-10 < k < 11$,	$-9 < k < 9$,	$-6 < k < 8$,
	$-7 < l < 5$	$-11 < l < 11$	$-8 < l < 11$	$-11 < l < 15$	$-8 < l < 11$	$-7 < l < 10$
**Reflections collected**	1295	678	2051	2963	1524	850
**Independent reflections / R_{int}**	833 / 0.0329	452 / 0.0549	900 / 0.0742	2014 / 0.0395	968 / 0.0397	672 / 0.0508
**Refinement method**	Full matrix least squares on F^2					
**Data / restraints / parameters**	833 / 0 / 143	452 / 0 / 97	900 / 0 / 97	2014 / 0 / 137	968 / 0 / 97	672 / 0 / 97
**Goodness of fit on F^2**	1.625	1.122	1.109	1.167	1.124	1.044
**Final R indices [$I > 2\sigma(I)$], R_1 / wR_2**	0.1364 / 0.3472	0.0811 / 0.2083	0.0884 / 0.2476	0.0555 / 0.1277	0.0628 / 0.1635	0.0728 / 0.1822
**R indices (all data), R_1 / wR_2**	0.1481 / 0.3632	0.1086 / 0.2280	0.0961 / 0.2599	0.0741 / 0.1399	0.0759 / 0.1740	0.0892 / 0.2020
**Largest diff. peak /hole (e·Å^{-3})**	1.178 / -1.421	0.700 / -0.597	1.397 / -0.895	0.800 / -0.672	0.784 / -0.775	0.644 / -0.631
**CSD number**	1860556	1860557	1860558	1860559	1860560	1860561
**Experimental details (beamline, wavelength, detector, beam size)**	P02.2, λ = 0.29004 Å, MAR345dtb, 2.5(H)x1.4(V) μm^2		ID09A, λ = 0.41273 Å, MAR555, 10(H)x10(V) μm^2	P02.2, λ = 0.28965 Å, Perkin Elmer XRD1621, 7.5(H)x3.5(V) μm^2		
**Data collection (ω range, step size)**	from -26 to +26°, 1° step		from -40 to +40°, 1° step*	from -40 to +29°, 0.25° step		

* *hkl* file was combined from two datasets collected at different orientations of the DAC in order to increase data completeness

Supplementary Table 4. Atomic coordinates and equivalent isotropic displacement parameters of coesite-III at 27.9(5) GPa

**Atom**	***x***	***y***	***z***	U_{iso} (Å^2)
Si1	0.4846(15)	0.0964(5)	0.892(3)	0.0166(12)
Si2	0.4684(16)	0.1068(6)	0.477(3)	0.0193(13)
Si3	0.2721(15)	0.2645(6)	0.279(3)	0.0170(12)
Si4	0.0798(15)	0.7676(5)	0.587(3)	0.0173(12)
Si5	0.4783(15)	0.7478(5)	0.076(3)	0.0163(12)
Si6	0.2071(16)	0.5509(6)	0.750(3)	0.0189(13)
Si7	0.2403(16)	0.5769(6)	0.172(3)	0.0179(12)
Si8	0.4074(15)	0.4189(5)	0.362(3)	0.0145(12)
Si9	0.1482(17)	0.3973(6)	0.747(3)	0.0181(12)
Si10	0.0861(15)	0.7682(6)	0.184(3)	0.0169(12)
Si11	0.1106(15)	0.0747(5)	0.004(3)	0.0172(13)
Si12	0.1415(16)	0.9180(6)	0.636(3)	0.0189(12)
O1	0.0000	0.0000	0.5000	0.053(7)
O2	0.0000	0.0000	0.0000	0.021(4)
O3	0.489(4)	0.0633(14)	0.679(8)	0.017(3)
O4	0.207(4)	0.0957(14)	0.253(8)	0.018(3)
O5	0.246(4)	0.9047(16)	0.900(8)	0.027(3)
O6	0.027(4)	0.1448(13)	0.483(7)	0.018(3)
O7	0.469(4)	0.1995(14)	0.478(8)	0.021(3)
O8	0.374(4)	0.1845(13)	0.818(7)	0.015(2)
O9	0.072(4)	0.2820(14)	0.331(8)	0.022(3)
O10	0.279(4)	0.7352(13)	0.133(7)	0.017(2)
O11	0.328(4)	0.3264(13)	0.815(7)	0.016(2)
O12	0.495(4)	0.4133(14)	0.617(7)	0.019(3)
O13	0.243(4)	0.6004(14)	0.953(7)	0.018(3)
O14	0.060(4)	0.4234(15)	0.493(8)	0.021(3)
O15	0.196(4)	0.4883(14)	0.226(7)	0.020(3)
O16	0.273(5)	0.4640(16)	0.884(9)	0.024(3)
O17	0.369(4)	0.5597(15)	0.645(8)	0.027(3)
O18	0.368(4)	0.3422(14)	0.267(7)	0.016(2)
O19	0.040(4)	0.6236(14)	0.183(7)	0.018(3)
O20	0.182(4)	0.2315(15)	0.054(8)	0.019(3)
O21	0.348(4)	0.7660(15)	0.792(8)	0.019(3)
O22	0.104(4)	0.7266(15)	0.403(8)	0.018(3)
O23	0.084(4)	0.8604(14)	0.180(7)	0.020(3)
O24	0.355(5)	0.9180(16)	0.601(9)	0.027(3)
O25	0.324(4)	0.0511(14)	0.964(7)	0.020(3)

*

Supplementary Table 5. Atomic coordinates and equivalent isotropic displacement parameters of coesite-IV at 35.9(7) GPa

Atom	*x*	*y*	*z*	U_{iso} (Å^2)
**Si1**	0.343(2)	0.6470(19)	0.4574(5)	0.0108(11)
**Si2**	0.1921(19)	0.5167(19)	0.9919(5)	0.0113(12)
**Si3**	0.1029(19)	0.3115(18)	0.6505(5)	0.0093(11)
**Si4**	0.496(2)	0.330(2)	0.2676(5)	0.0121(12)
**Si5**	0.349(2)	0.1278(18)	0.9200(5)	0.0099(11)
**Si6**	0.199(2)	0.0597(19)	0.4397(5)	0.0094(12)
**Si7**	0.039(2)	0.8616(18)	0.8375(5)	0.0102(11)
**Si8**	0.2806(19)	0.7717(18)	0.1392(5)	0.0091(12)
**O1**	0.328(5)	0.840(4)	0.5202(12)	0.011(3)
**O2**	0.470(5)	0.791(4)	0.2580(13)	0.009(2)
**O3**	0.149(5)	0.680(4)	0.3230(12)	0.008(3)
**O4**	0.174(5)	0.517(4)	0.6127(13)	0.010(2)
**O5**	0.450(5)	0.429(5)	0.4144(13)	0.010(2)
**O6**	0.097(4)	0.745(4)	0.0412(12)	0.008(2)
**O7**	0.363(4)	0.514(4)	0.1269(12)	0.008(2)
**O8**	0.039(5)	0.382(5)	0.1363(13)	0.014(3)
**O9**	0.176(5)	0.020(4)	0.1006(13)	0.010(3)
**O10**	0.366(6)	0.138(6)	0.2878(14)	0.013(3)
**O11**	0.164(5)	0.266(4)	0.4901(13)	0.012(3)
**O12**	0.179(5)	0.101(4)	0.7975(12)	0.008(2)
**O13**	0.274(5)	0.354(4)	0.9001(13)	0.008(2)
**O14**	0.287(5)	0.721(4)	0.8186(13)	0.011(3)
**O15**	0.000(5)	0.957(4)	0.6403(13)	0.010(3)
**O16**	0.435(4)	0.866(4)	0.9701(11)	0.007(3)

Supplementary Table 6. Atomic coordinates and equivalent isotropic displacement parameters of coesite-IV at 40.2(7) GPa

Atom	*x*	*y*	*z*	U_{iso} (Å^2)
**Si1**	0.3439(3)	0.6473(5)	0.4572(4)	0.0182(6)
**Si2**	0.1944(3)	0.5151(5)	0.9943(4)	0.0187(6)
**Si3**	0.0987(3)	0.3126(5)	0.6489(4)	0.0177(5)
**Si4**	0.4972(3)	0.3191(5)	0.2729(5)	0.0189(6)
**Si5**	0.3482(3)	0.1279(5)	0.9184(4)	0.0186(6)
**Si6**	0.1974(3)	0.0636(5)	0.4430(5)	0.0205(6)
**Si7**	0.0402(3)	0.8618(5)	0.8379(4)	0.0189(6)
**Si8**	0.2856(3)	0.7706(5)	0.1386(4)	0.0179(6)
**O1**	0.3286(8)	0.8467(13)	0.5239(12)	0.0199(11)
**O2**	0.4680(8)	0.7828(12)	0.2581(11)	0.0186(11)
**O3**	0.1504(8)	0.6810(12)	0.3239(11)	0.0162(11)
**O4**	0.1819(8)	0.5214(13)	0.6163(11)	0.0189(11)
**O5**	0.4569(8)	0.4303(12)	0.4139(11)	0.0198(11)
**O6**	0.0930(8)	0.7417(13)	0.0426(12)	0.0199(11)
**O7**	0.3719(8)	0.5145(13)	0.1300(11)	0.0206(12)
**O8**	0.0359(8)	0.3746(12)	0.1394(11)	0.0197(11)
**O9**	0.1836(8)	0.0244(13)	0.0997(11)	0.0171(11)
**O10**	0.3695(8)	0.1405(13)	0.2909(12)	0.0205(12)
**O11**	0.1565(8)	0.2678(13)	0.4811(11)	0.0208(12)
**O12**	0.1755(8)	0.0949(13)	0.7949(11)	0.0205(12)
**O13**	0.2756(8)	0.3583(13)	0.8982(11)	0.0210(12)
**O14**	0.2850(8)	0.7174(13)	0.8215(11)	0.0193(11)
**O15**	0.0011(9)	0.9625(13)	0.6389(11)	0.0215(12)
**O16**	0.4434(7)	0.8695(12)	0.9673(11)	0.0162(10)

Supplementary Table 7. Atomic coordinates and equivalent isotropic displacement parameters of coesite-IV at 44.2(4) GPa

**Atom**	***x***	***y***	***z***	U_{iso} **or** U_{eq}***(Å^2)**
**Si1**	0.34502(13)	0.6487(2)	0.4581(2)	0.0085(9)
**Si2**	0.19452(14)	0.5156(2)	0.9955(2)	0.0091(8)
**Si3**	0.09874(14)	0.3143(2)	0.6504(2)	0.0093(8)
**Si4**	0.49713(14)	0.3179(2)	0.2743(2)	0.0107(9)
**Si5**	0.34835(13)	0.1267(2)	0.91817(19)	0.0071(8)
**Si6**	0.19587(14)	0.0639(2)	0.4449(2)	0.0084(8)
**Si7**	0.04054(14)	0.8622(2)	0.83772(19)	0.0082(8)
**Si8**	0.28605(13)	0.7703(2)	0.13940(19)	0.0072(8)
**O1**	0.3339(4)	0.8483(6)	0.5264(6)	0.0092(4)
**O2**	0.4673(3)	0.7872(5)	0.2598(5)	0.0064(3)
**O3**	0.1507(3)	0.6814(6)	0.3255(5)	0.0064(3)
**O4**	0.1810(4)	0.5259(6)	0.6175(5)	0.0082(4)
**O5**	0.4556(4)	0.4311(6)	0.4144(5)	0.0075(3)
**O6**	0.0932(3)	0.7422(6)	0.0436(5)	0.0070(3)
**O7**	0.3731(3)	0.5161(6)	0.1294(5)	0.0077(3)
**O8**	0.0352(3)	0.3743(6)	0.1397(5)	0.0066(3)
**O9**	0.1840(3)	0.0257(5)	0.1007(5)	0.0060(3)
**O10**	0.3656(4)	0.1420(6)	0.2882(5)	0.0084(4)
**O11**	0.1559(4)	0.2709(6)	0.4825(5)	0.0097(4)
**O12**	0.1769(3)	0.0947(5)	0.7949(5)	0.0064(3)
**O13**	0.2766(3)	0.3608(6)	0.8989(5)	0.0075(3)
**O14**	0.2852(3)	0.7178(5)	0.8214(5)	0.0071(3)
**O15**	0.0030(3)	0.9632(6)	0.6383(5)	0.0069(3)
**O16**	0.4459(3)	0.8719(5)	0.9687(5)	0.0063(3)

*U_{eq} is defined as one third of the trace of the orthogonalized U_{ij} tensor

Supplementary Table 8. Atomic coordinates and equivalent isotropic displacement parameters of coesite-IV at 49.3(8) GPa

**Atom**	***x***	***y***	***z***	U_{iso} (Å^2)
**Si1**	0.3455(3)	0.6507(4)	0.4585(4)	0.0117(4)
**Si2**	0.1950(3)	0.5148(4)	0.9956(3)	0.0126(4)
**Si3**	0.0987(3)	0.3168(4)	0.6523(4)	0.0123(4)
**Si4**	0.4973(3)	0.3148(4)	0.2769(3)	0.0132(4)
**Si5**	0.3484(3)	0.1254(4)	0.9178(3)	0.0120(4)
**Si6**	0.1946(3)	0.0642(4)	0.4476(4)	0.0130(4)
**Si7**	0.0399(3)	0.8619(4)	0.8374(3)	0.0120(4)
**Si8**	0.2877(3)	0.7692(4)	0.1399(3)	0.0118(4)
**O1**	0.3400(7)	0.8503(11)	0.5268(10)	0.0139(8)
**O2**	0.4681(7)	0.7891(11)	0.2613(9)	0.0125(8)
**O3**	0.1515(7)	0.6828(10)	0.3278(9)	0.0114(8)
**O4**	0.1791(7)	0.5305(11)	0.6189(9)	0.0149(9)
**O5**	0.4558(7)	0.4323(10)	0.4141(8)	0.0124(8)
**O6**	0.0918(7)	0.7407(10)	0.0437(9)	0.0118(8)
**O7**	0.3741(7)	0.5149(11)	0.1283(9)	0.0122(8)
**O8**	0.0375(7)	0.3760(11)	0.1414(9)	0.0137(8)
**O9**	0.1853(7)	0.0256(10)	0.1019(9)	0.0119(8)
**O10**	0.3615(7)	0.1416(11)	0.2885(9)	0.0142(8)
**O11**	0.1576(7)	0.2732(11)	0.4817(10)	0.0163(9)
**O12**	0.1755(7)	0.0945(10)	0.7954(9)	0.0125(8)
**O13**	0.2752(7)	0.3620(11)	0.8974(9)	0.0143(8)
**O14**	0.2844(7)	0.7172(10)	0.8208(9)	0.0122(8)
**O15**	0.0041(7)	0.9632(11)	0.6376(9)	0.0134(8)
**O16**	0.4477(6)	0.8710(10)	0.9698(9)	0.0114(8)

Supplementary Table 9. Atomic coordinates and equivalent isotropic displacement parameters of coesite-V at 56.7(9) GPa

Atom	*x*	*y*	*z*	U_{iso} (Å^2)
**Si1**	0.3365(5)	0.6559(8)	0.4581(6)	0.0193(6)
**Si2**	0.1973(5)	0.5159(8)	0.9821(6)	0.0197(6)
**Si3**	0.1089(5)	0.3381(8)	0.6855(6)	0.0197(6)
**Si4**	0.4915(5)	0.3111(8)	0.2781(6)	0.0197(6)
**Si5**	0.3497(5)	0.1102(7)	0.9148(5)	0.0183(6)
**Si6**	0.1962(5)	0.0677(8)	0.4659(6)	0.0198(6)
**Si7**	0.0339(4)	0.8628(7)	0.8335(5)	0.0192(6)
**Si8**	0.2911(4)	0.7684(8)	0.1401(6)	0.0187(6)
**O1**	0.3597(12)	0.865(2)	0.5358(15)	0.0205(14)
**O2**	0.4662(12)	0.7976(19)	0.2581(14)	0.0187(13)
**O3**	0.1487(11)	0.6777(18)	0.3141(14)	0.0176(12)
**O4**	0.1438(12)	0.578(2)	0.6027(15)	0.0215(14)
**O5**	0.4721(12)	0.452(2)	0.3980(16)	0.0201(14)
**O6**	0.0963(12)	0.741(2)	0.0355(14)	0.0204(14)
**O7**	0.3843(11)	0.5141(19)	0.1218(14)	0.0190(13)
**O8**	0.0472(11)	0.3807(18)	0.1354(14)	0.0180(13)
**O9**	0.1930(11)	0.0274(19)	0.0992(14)	0.0189(13)
**O10**	0.3381(12)	0.152(2)	0.2944(15)	0.0203(13)
**O11**	0.2073(12)	0.264(2)	0.5352(17)	0.0226(15)
**O12**	0.1670(12)	0.074(2)	0.8030(14)	0.0194(13)
**O13**	0.2460(11)	0.3524(19)	0.8637(14)	0.0177(13)
**O14**	0.2825(11)	0.7095(19)	0.8151(15)	0.0188(13)
**O15**	0.0016(11)	0.9501(19)	0.6301(14)	0.0184(13)
**O16**	0.4621(11)	0.8572(19)	0.9721(15)	0.0177(13)

Supplementary Table 10. Comparison between of lattice parameters of coesite-IV and coesite-V observed experimentally and determined by *ab initio* calculations. As starting model for *ab initio* relaxation experimental data were used. The structural relaxation was done with the conjugate-gradient algorithm as implemented in VASP. The AM05 functional was used for the exchange and correlation description.

		**Pressure, GPa**	***V*, Å^3**	***a*, Å**	***b*, Å**	***c*, Å**	**α, °**	**β, °**	**γ, °**
**coesite-IV**	exp.	40.2(7)	368.5(2)	6.5989(8)	7.000(2)	8.648(3)	69.14(3)	83.15(2)	81.80(2)
	calc.	39	368.5011	6.60893	7.02291	8.62297	68.9146	82.8546	81.8671
**coesite-IV**	exp.	35.9(7)	366.1(7)	6.524(9)	6.942(9)	8.674(5)	70.07(8)	84.05(7)	83.6(1)
	calc.	41	366.1221	6.59406	7.00445	8.60456	69.0070	82.8629	81.8301
**coesite-IV**	exp.	44.2(4)	357.83(5)	6.5586(4)	6.9029(7)	8.5429(8)	69.599(9)	83.035(6)	81.933(6)
	calc.	48	357.8274	6.53660	6.93415	8.53955	69.5695	82.9673	81.6966
**coesite-IV**	exp.	49.3(8)	352.35(9)	6.5175(8)	6.875(1)	8.494(2)	69.61(2)	83.29(1)	81.99(1)
	calc.	55	352.3491	6.42213	6.84399	8.52062	72.4568	83.9784	81.3844
**coesite-V**	exp.	56.7(9)	342.6(2)	6.403(2)	6.768(2)	8.400(3)	72.40(3)	84.02(3)	81.64(3)
	calc.	57	342.5716	6.37189	6.76641	8.46603	72.2540	83.6899	80.9525

Supplementary Table 11. Bulk modules of different silica phases as obtained by *ab initio* calculations.

	**K_0, GPa**	**K'_0**
**stishovite/$CaCl_2$**	298.90	3.66
**seifertite**	310.14	4.09
**coesite-IV**	168.52	3.34
**coesite-V**	185.26	3.10

Supplementary references

1. Černok, A. *et al.* High-pressure crystal chemistry of coesite-I and its transition to coesite-II. *Zeitschrift für Krist. - Cryst. Mater.* **229,** 761–773 (2014).

2. Momma, K. & Izumi, F. VESTA 3 for three-dimensional visualization of crystal, volumetric and morphology data. *J. Appl. Crystallogr.* **44,** 1272–1276 (2011).

3. Robinson, K., Gibbs, G. V. & Ribbe, P. H. Quadratic Elongation: A Quantitative Measure of Distortion in Coordination Polyhedra. *Science* **172,** 567–570 (1971).

4. Yamanaka, T., Fukuda, T. & Mimaki, J. Bonding character of SiO_2 stishovite under high pressures up to 30 GPa. *Phys. Chem. Miner.* **29,** 633–641 (2002).

5. Černok, A. *et al.* Compressional pathways of α-cristobalite, structure of cristobalite X-I, and towards the understanding of seifertite formation. *Nat. Commun.* **8,** 15647 (2017).

6. Angel, R. J., Ross, N. L., Seifert, F. & Fliervoet, T. F. Structural characterization of pentacoordinate silicon in a calcium silicate. *Nature* **384,** 441–444 (1996).

7. Liu, X. *et al.* Anhydrous ringwoodites in the mantle transition zone: Their bulk modulus, solid solution behavior, compositional variation, and sound velocity feature. *Solid Earth Sci.* **1,** 28–47 (2016).

8. Finkelstein, G. J., Dera, P. K. & Duffy, T. S. High-pressure phases of cordierite from single-crystal X-ray diffraction to 15 GPa. *Am. Mineral.* **100,** 1821–1833 (2015).

9. Ballaran, T. B. *et al.* Effect of chemistry on the compressibility of silicate perovskite in the lower mantle. *Earth Planet. Sci. Lett.* **333,** 181–190 (2012).

10. Finkelstein, G. J., Dera, P. K. & Duffy, T. S. Phase transitions in orthopyroxene (En_{90}) to 49 GPa from single-crystal X-ray diffraction. *Phys. Earth Planet. Inter.* **244,** 78–86 (2015).

11. Hu, Y., Kiefer, B., Bina, C. R., Zhang, D. & Dera, P. K. High-Pressure γ-$CaMgSi_2O_6$: Does penta-coordinated silicon exist in the Earth's mantle? *Geophys. Res. Lett.* **44,** 11,340-11,348 (2017).

12. Glazyrin, K. *et al.* Critical behavior of Mg_{1-x} Fe_xO at the pressure-induced iron spin-state crossover. *Phys. Rev. B* **95,** (2017).

13. Sanloup, C. *et al.* Structural change in molten basalt at deep mantle conditions. *Nature* **503,** 104–107 (2013).

14. Prescher, C. *et al.* Beyond sixfold coordinated Si in SiO_2 glass at ultrahigh pressures. *Proc. Natl. Acad. Sci.* **114,** 10041–10046 (2017).

15. Kono, Y., Shibazaki, Y., Kenney-Benson, C., Wang, Y. & Shen, G. Pressure-induced structural change in $MgSiO_3$ glass at pressures near the Earth's core–mantle boundary. *Proc. Natl. Acad. Sci.* **115,** 1742–1747 (2018).

16. Černok, A. *et al.* Pressure-induced phase transitions in coesite. *Am. Mineral.* **99,** 755–763 (2014).

17. Angel, R. J., Allan, D. R., Miletich, R. & Finger, L. W. The Use of Quartz as an Internal Pressure Standard in High-Pressure Crystallography. *J. Appl. Crystallogr.* **30,** 461–466 (1997).

18. Andrault, D., Angel, R. J., Mosenfelder, J. L. & Le Bihan, T. Equation of state of stishovite to lower mantle pressures. *Am. Mineral.* **88,** 301–307 (2003).

19. Grocholski, B., Shim, S.-H. & Prakapenka, V. B. Stability, metastability, and elastic properties of a dense silica polymorph, seifertite. *J. Geophys. Res. Solid Earth* **118,** 4745–4757 (2013).

20. Petitgirard, S. *et al.* SiO_2 Glass Density to Lower-Mantle Pressures. *Phys. Rev. Lett.* **119,** 215701 (2017).
