# Supplementary material for: Metastable silica high pressure polymorphs as structural proxies of deep Earth silicate melts
Source: Nat Commun. 2018 Nov 15;9:4789. doi: 10.1038/s41467-018-07265-z (PMC6237875; doi:10.1038/s41467-018-07265-z)
Supplement: Supplementary file 2 — Description of Additional Supplementary Files [file 41467_2018_7265_MOESM2_ESM.docx]

**Description of Additional Supplementary Files**

File Name: Supplementary Movie 1

Description: Transformational path from coesite-IV to coesite-V inferred from first principles calculations.

**File Name**: Supplementary Data 1

**Description**: Crystal structure of coesite-III at 28 GPa”.

**File Name**: Supplementary Data 2

**Description**: Crystal structure of coesite-IV at 36 GPa.

**File Name**: Supplementary Data 3

**Description**: Crystal structure of coesite-IV at 40 GPa.

**File Name**: Supplementary Data 4

**Description**: Crystal structure of coesite-IV at 44 GPa.

**File Name**: Supplementary Data 5

**Description**: Crystal structure of coesite-IV at 49 GPa.

**File Name**: Supplementary Data 6

**Description**: Crystal structure of coesite-V at 57 GPa.

**File Name**: Supplementary Data 7

**Description**: Checkcif report of crystal structure of coesite-III at 28 GPa.

**File Name**: Supplementary Data 8

**Description**: Checkcif report of crystal structure of coesite-IV at 36 GPa.

**File Name**: Supplementary Data 9

**Description**: Checkcif report of crystal structure of coesite-IV at 40 GPa.

**File Name**: Supplementary Data 10

**Description**: Checkcif report of crystal structure of coesite-IV at 44 GPa.

**File Name**: Supplementary Data 11

**Description**: Checkcif report of crystal structure of coesite-IV at 49 GPa.

**File Name**: Supplementary Data 12

**Description**: Checkcif report of crystal structure of coesite-V at 57 GPa.

**File Name**: Featured image

**Description**: Crystal structure of coesite at high pressure.
