# Supplementary material for: Metastable silica high pressure polymorphs as structural proxies of deep Earth silicate melts
Source: Nat Commun. 2018 Nov 15;9:4789. doi: 10.1038/s41467-018-07265-z (PMC6237875; doi:10.1038/s41467-018-07265-z)

checkCIF/PLATON report

Structure factors have been supplied for datablock(s) Coesite-III_27.9GPa

THIS REPORT IS FOR GUIDANCE ONLY. IF USED AS PART OF A REVIEW PROCEDURE FOR PUBLICATION, IT SHOULD NOT REPLACE THE EXPERTISE OF AN EXPERIENCED CRYSTALLOGRAPHIC REFEREE.

No syntax errors found. CIF dictionary Interpreting this report

Datablock: Coesite-III_27.9GPa

```
Bond precision:  Si- O = 0.0401 A        Wavelength=0.29004

Cell:           a=6.571(3)        b=17.790(7)       c=6.766(4)
                alpha=82.14(4)    beta=120.81(6)    gamma=87.13(3)
Temperature:    296 K

                Calculated                Reported
Volume          666.2(7)                  666.2(6)
Space group     P -1                      P -1
Hall group      -P 1                      -P 1
Moiety formula  O24 Si12                  O2 Si
Sum formula     O24 Si12                  O2 Si
Mr              721.08                    60.09
Dx,g cm-3       3.595                     3.595
Z               2                         24
Mu (mm-1)       0.149                     0.149
F000            720.0                     720.0
F000’           720.06
h,k,lmax        8,23,8                    8,23,7
Nref            3289                      833
Tmin,Tmax                                 0.172,1.000
Tmin’

Correction method= # Reported T Limits: Tmin=0.172 Tmax=1.000
AbsCorr = MULTI-SCAN

Data completeness= 0.253          Theta(max)= 11.146

R(reflections)= 0.1364( 681)      wR2(reflections)= 0.3632( 833)

S = 1.625                 Npar= 143
```

```
The following ALERTS were generated. Each ALERT has the format
       test-name_ALERT_alert-type_alert-level.
Click on the hyperlinks for more details of the test.
```

Alert level A

```
ATOM007_ALERT_1_A  _atom_site_aniso_label is missing
            Unique label identifying the atom site.
```

Author Response: Due to incompleteness of the dataset thermal parameters were refined in isotropic approximation.

```
PLAT029_ALERT_3_A _diffrn_measured_fraction_theta_full value Low .      0.253 Why?
```

Author Response: The dataset was incomplete since the data were collected in a diamond anvil cell metallic body of which shadows more than 60% of the reflections.

```
PLAT088_ALERT_3_A Poor Data / Parameter Ratio ....................       5.83 Note
```

Author Response: Poor data/parameter ratio is due to low symmetry of the structure (P-1), big amount of the parameters to refine (27 atoms) and incompleteness of the dataset (measurement in a diamond anvil cell).

```
PLAT910_ALERT_3_A Missing # of FCF Reflection(s) Below Theta(Min).         52 Note
```

Author Response: Certain part of the reflections is missing due to geometry of the experiment. The metallic body of the diamond anvil cell absorbs more than 60% of the reflections.

Alert level B

```
DIFMN02_ALERT_2_B  The minimum difference density is < -0.1*ZMAX*1.00
            _refine_diff_density_min given =     -1.421
            Test value =     -1.400
```

Author Response: Due to partial overlap of the reflections with those belonging to low-pressure phase (coesite-II) and poor data/parameter ratio.

```
PLAT084_ALERT_3_B High wR2 Value (i.e. > 0.25) ..................       0.36 Report
```

Author Response: Due to partial overlap of the reflections with those belonging to low-pressure phase (coesite-II).

```
PLAT098_ALERT_2_B Large Reported Min.  (Negative) Residual Density      -1.42 eA-3
```

Author Response: Due to partial overlap of the reflections with those belonging to low-pressure phase (coesite-II) and poor data/parameter ratio.

```
PLAT911_ALERT_3_B Missing FCF Refl Between Thmin & STh/L=    0.600       1717 Report
```

Author Response: Certain part of the reflections is missing due to geometry of the experiment. The metallic body of the diamond anvil cell absorbs more than 60% of the reflections.

Alert level C

```
DIFMN03_ALERT_1_C  The minimum difference density is < -0.1*ZMAX*0.75
            The relevant atom site should be identified.
DIFMX02_ALERT_1_C  The maximum difference density is > 0.1*ZMAX*0.75
            The relevant atom site should be identified.
PLAT082_ALERT_2_C High R1 Value ..................................       0.14 Report
PLAT097_ALERT_2_C Large Reported Max.  (Positive) Residual Density       1.18 eA-3
PLAT155_ALERT_4_C The Triclinic Unitcell is NOT Reduced ..........     Please Do !
PLAT906_ALERT_3_C Large K Value in the Analysis of Variance ......      4.738 Check
PLAT913_ALERT_3_C Missing # of Very Strong Reflections in FCF ....          9 Note
PLAT975_ALERT_2_C Check Calcd Resid. Dens.  0.65A   From O3              1.04 eA-3
PLAT976_ALERT_2_C Check Calcd Resid. Dens.  0.92A   From O21            -0.96 eA-3
```

Alert level G

```
ABSMU01_ALERT_1_G  Calculation of _exptl_absorpt_correction_mu
                not performed for this radiation type.
PLAT004_ALERT_5_G Polymeric Structure Found with Maximum Dimension          3 Info
PLAT012_ALERT_1_G N.O.K.   _shelx_res_checksum Found in CIF ......     Please Check
PLAT042_ALERT_1_G Calc. and Reported MoietyFormula Strings  Differ     Please Check
PLAT045_ALERT_1_G Calculated and Reported Z Differ by a Factor ...       0.08 Check
PLAT072_ALERT_2_G SHELXL First  Parameter in WGHT  Unusually Large       0.20 Report
PLAT396_ALERT_2_G Deviating Si-O-Si   Angle From 150 for O1             180.0 Degree
PLAT396_ALERT_2_G Deviating Si-O-Si   Angle From 150 for O2             180.0 Degree
PLAT396_ALERT_2_G Deviating Si-O-Si   Angle From 150 for O3             126.9 Degree
PLAT396_ALERT_2_G Deviating Si-O-Si   Angle From 150 for O4             136.9 Degree
PLAT396_ALERT_2_G Deviating Si-O-Si   Angle From 150 for O5             129.4 Degree
PLAT396_ALERT_2_G Deviating Si-O-Si   Angle From 150 for O6             120.5 Degree
PLAT396_ALERT_2_G Deviating Si-O-Si   Angle From 150 for O7             125.1 Degree
PLAT396_ALERT_2_G Deviating Si-O-Si   Angle From 150 for O8             124.6 Degree
PLAT396_ALERT_2_G Deviating Si-O-Si   Angle From 150 for O9             136.2 Degree
PLAT396_ALERT_2_G Deviating Si-O-Si   Angle From 150 for O11            171.4 Degree
PLAT396_ALERT_2_G Deviating Si-O-Si   Angle From 150 for O12            128.1 Degree
PLAT396_ALERT_2_G Deviating Si-O-Si   Angle From 150 for O13            130.1 Degree
PLAT396_ALERT_2_G Deviating Si-O-Si   Angle From 150 for O14            135.0 Degree
PLAT396_ALERT_2_G Deviating Si-O-Si   Angle From 150 for O15            122.6 Degree
PLAT396_ALERT_2_G Deviating Si-O-Si   Angle From 150 for O16            120.9 Degree
PLAT396_ALERT_2_G Deviating Si-O-Si   Angle From 150 for O19            135.1 Degree
PLAT396_ALERT_2_G Deviating Si-O-Si   Angle From 150 for O20            134.8 Degree
PLAT396_ALERT_2_G Deviating Si-O-Si   Angle From 150 for O21            132.5 Degree
PLAT396_ALERT_2_G Deviating Si-O-Si   Angle From 150 for O22            124.6 Degree
PLAT396_ALERT_2_G Deviating Si-O-Si   Angle From 150 for O23            127.6 Degree
PLAT396_ALERT_2_G Deviating Si-O-Si   Angle From 150 for O25            135.2 Degree
PLAT432_ALERT_2_G Short Inter X...Y Contact  Si1      ..O25              2.67 Ang.
PLAT432_ALERT_2_G Short Inter X...Y Contact  Si1      ..O2               3.39 Ang.
PLAT432_ALERT_2_G Short Inter X...Y Contact  Si1      ..Si1              3.49 Ang.
PLAT432_ALERT_2_G Short Inter X...Y Contact  Si2      ..O3               3.38 Ang.
PLAT432_ALERT_2_G Short Inter X...Y Contact  Si3      ..Si5              3.56 Ang.
PLAT432_ALERT_2_G Short Inter X...Y Contact  Si5      ..O20              2.81 Ang.
PLAT432_ALERT_2_G Short Inter X...Y Contact  Si11     ..O3               3.11 Ang.
PLAT432_ALERT_2_G Short Inter X...Y Contact  Si11     ..O5               3.37 Ang.
```

```
PLAT432_ALERT_2_G Short Inter X...Y Contact  Si12     ..Si12            3.14 Ang.
PLAT793_ALERT_4_G Model has Chirality at Si3        (Centro SPGR)          R Verify
PLAT793_ALERT_4_G Model has Chirality at Si5        (Centro SPGR)          R Verify
PLAT793_ALERT_4_G Model has Chirality at Si7        (Centro SPGR)          S Verify
PLAT793_ALERT_4_G Model has Chirality at Si8        (Centro SPGR)          R Verify
PLAT912_ALERT_4_G Missing # of FCF Reflections Above STh/L=  0.600        571 Note
PLAT933_ALERT_2_G Number of OMIT Records in Embedded .res File ...          5 Note
```

```
   4 ALERT level A = Most likely a serious problem - resolve or explain
   4 ALERT level B = A potentially serious problem, consider carefully
   9 ALERT level C = Check. Ensure it is not caused by an omission or oversight
  42 ALERT level G = General information/check it is not something unexpected

   7 ALERT type 1 CIF construction/syntax error, inconsistent or missing data
  38 ALERT type 2 Indicator that the structure model may be wrong or deficient
   7 ALERT type 3 Indicator that the structure quality may be low
   6 ALERT type 4 Improvement, methodology, query or suggestion
   1 ALERT type 5 Informative message, check
```

It is advisable to attempt to resolve as many as possible of the alerts in all categories. Often the minor alerts point to easily fixed oversights, errors and omissions in your CIF or refinement strategy, so attention to these fine details can be worthwhile. In order to resolve some of the more serious problems it may be necessary to carry out additional measurements or structure refinements. However, the purpose of your study may justify the reported deviations and the more serious of these should normally be commented upon in the discussion or experimental section of a paper or in the "special_details" fields of the CIF. checkCIF was carefully designed to identify outliers and unusual parameters, but every test has its limitations and alerts that are not important in a particular case may appear. Conversely, the absence of alerts does not guarantee there are no aspects of the results needing attention. It is up to the individual to critically assess their own results and, if necessary, seek expert advice.

Publication of your CIF in IUCr journals

A basic structural check has been run on your CIF. These basic checks will be run on all CIFs submitted for publication in IUCr journals (*Acta Crystallographica*, *Journal of Applied Crystallography*, *Journal of Synchrotron Radiation*); however, if you intend to submit to *Acta Crystallographica Section C* or *E* or *IUCrData*, you should make sure that full publication checks are run on the final version of your CIF prior to submission.

Publication of your CIF in other journals

Please refer to the *Notes for Authors* of the relevant journal for any special instructions relating to CIF submission.

PLATON version of 30/01/2018; check.def file version of 30/01/2018

Datablock Coesite-III_27.9GPa - ellipsoid plot

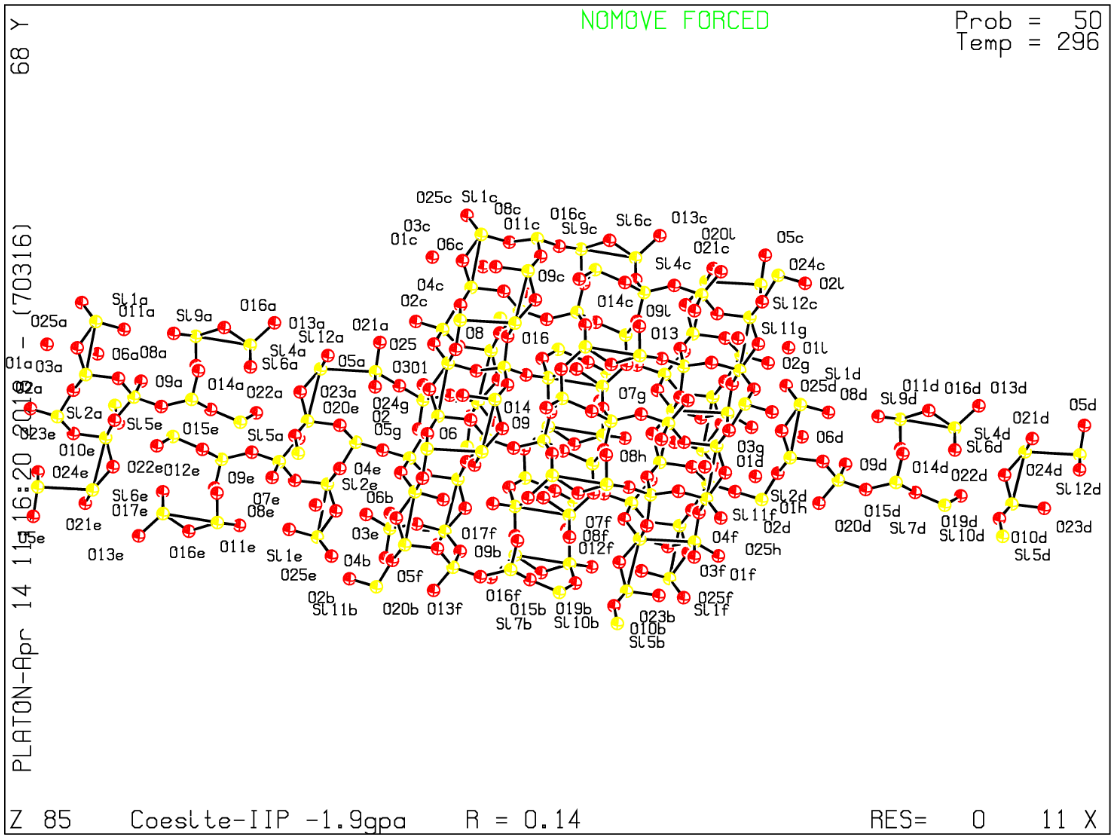

Supplement: Supplementary file 10 — Supplementary Data 7 [file 41467_2018_7265_MOESM10_ESM.pdf]
