# Supplementary material for: Metastable silica high pressure polymorphs as structural proxies of deep Earth silicate melts
Source: Nat Commun. 2018 Nov 15;9:4789. doi: 10.1038/s41467-018-07265-z (PMC6237875; doi:10.1038/s41467-018-07265-z)

checkCIF/PLATON report

Structure factors have been supplied for datablock(s) Coesite-IV_44.18GPa

THIS REPORT IS FOR GUIDANCE ONLY. IF USED AS PART OF A REVIEW PROCEDURE FOR PUBLICATION, IT SHOULD NOT REPLACE THE EXPERTISE OF AN EXPERIENCED CRYSTALLOGRAPHIC REFEREE.

No syntax errors found.   CIF dictionary   Interpreting this report

Datablock: Coesite-IV_44.18GPa

```
Bond precision:  Si- O = 0.0038 A        Wavelength=0.28965

Cell:          a=6.5586(4)       b=6.9029(7)      c=8.5429(8)
               alpha=69.599(9)   beta=83.035(6)   gamma=81.933(6)
Temperature:   296 K

                Calculated                Reported
Volume          357.83(6)                 357.83(6)
Space group     P -1                      P -1
Hall group      -P 1                      -P 1
Moiety formula  O16 Si8                   O2 Si
Sum formula     O16 Si8                   O2 Si
Mr              480.72                    60.09
Dx,g cm-3       4.462                     4.462
Z               2                         16
Mu (mm-1)       0.185                     0.185
F000            480.0                     480.0
F000'           480.04
h,k,lmax        15,15,19                  14,11,15
Nref            9264                      2014
Tmin,Tmax                                 0.800,1.000
Tmin'

Correction method= # Reported T Limits: Tmin=0.800 Tmax=1.000
AbsCorr = MULTI-SCAN

Data completeness= 0.217          Theta(max)= 19.569

R(reflections)= 0.0555( 1562)     wR2(reflections)= 0.1399( 2014)

S = 1.167                  Npar= 137
```

The following ALERTS were generated. Each ALERT has the format
test-name_ALERT_alert-type_alert-level.
Click on the hyperlinks for more details of the test.

Alert level A

PLAT029_ALERT_3_A _diffrn_measured_fraction_theta_full value Low . 0.370 Why?

Author Response: The dataset was incomplete since the data were collected in a diamond anvil cell metallic body of which shadows more than 60% of the reflections.

Alert level B

PLAT911_ALERT_3_B Missing FCF Refl Between Thmin & STh/L= 0.600 808 Report

Author Response: Certain part of the reflections is missing due to geometry of the experiment. The metallic body of the diamond anvil cell absorbs more than 60% of the reflections.

Alert level C

```
PLAT230_ALERT_2_C Hirshfeld Test Diff for   Si1      --Si4_k     .        5.5 s.u.
PLAT230_ALERT_2_C Hirshfeld Test Diff for   Si2      --Si8_d     .        5.4 s.u.
PLAT230_ALERT_2_C Hirshfeld Test Diff for   Si4      --Si1_k     .        5.5 s.u.
PLAT230_ALERT_2_C Hirshfeld Test Diff for   Si8      --Si2_c     .        5.4 s.u.
PLAT250_ALERT_2_C Large U3/U1 Ratio for Average U(i,j) Tensor ....        2.6 Note
PLAT906_ALERT_3_C Large K Value in the Analysis of Variance ......      7.325 Check
PLAT910_ALERT_3_C Missing # of FCF Reflection(s) Below Theta(Min).          8 Note
```

Alert level G

```
ABSMU01_ALERT_1_G  Calculation of _exptl_absorpt_correction_mu
                not performed for this radiation type.
PLAT004_ALERT_5_G Polymeric Structure Found with Maximum Dimension          3 Info
PLAT012_ALERT_1_G N.O.K.   _shelx_res_checksum Found in CIF ......     Please Check
PLAT042_ALERT_1_G Calc. and Reported MoietyFormula Strings  Differ     Please Check
PLAT045_ALERT_1_G Calculated and Reported Z Differ by a Factor ...       0.13 Check
PLAT396_ALERT_2_G Deviating Si-O-Si   Angle From 150 for O4             127.4 Degree
PLAT396_ALERT_2_G Deviating Si-O-Si   Angle From 150 for O10            120.7 Degree
PLAT396_ALERT_2_G Deviating Si-O-Si   Angle From 150 for O11            131.1 Degree
PLAT432_ALERT_2_G Short Inter X...Y Contact  Si1      ..O15              3.20 Ang.
PLAT432_ALERT_2_G Short Inter X...Y Contact  Si4      ..O7               3.28 Ang.
PLAT432_ALERT_2_G Short Inter X...Y Contact  Si4      ..Si6              3.47 Ang.
PLAT432_ALERT_2_G Short Inter X...Y Contact  Si5      ..O9               3.10 Ang.
PLAT432_ALERT_2_G Short Inter X...Y Contact  Si5      ..O10              3.25 Ang.
PLAT432_ALERT_2_G Short Inter X...Y Contact  Si7      ..O3               3.11 Ang.
PLAT432_ALERT_2_G Short Inter X...Y Contact  Si7      ..Si8              3.13 Ang.
PLAT432_ALERT_2_G Short Inter X...Y Contact  Si7      ..O9               3.13 Ang.
PLAT432_ALERT_2_G Short Inter X...Y Contact  Si7      ..O6               3.21 Ang.
PLAT432_ALERT_2_G Short Inter X...Y Contact  Si8      ..O16              3.05 Ang.
PLAT432_ALERT_2_G Short Inter X...Y Contact  Si8      ..O15              3.34 Ang.
PLAT912_ALERT_4_G Missing # of FCF Reflections Above STh/L=  0.600       4858 Note
PLAT913_ALERT_3_G Missing # of Very Strong Reflections in FCF ....          2 Note
PLAT933_ALERT_2_G Number of OMIT Records in Embedded .res File ...         19 Note
```

```
PLAT951_ALERT_5_G Calculated (ThMax) and CIF-Reported Kmax Differ           4 Units
PLAT952_ALERT_5_G Calculated (ThMax) and CIF-Reported Lmax Differ           4 Units
PLAT957_ALERT_1_G Calculated (ThMax) and Actual (FCF) Kmax Differ           4 Units
PLAT958_ALERT_1_G Calculated (ThMax) and Actual (FCF) Lmax Differ           4 Units
```

```
  1 ALERT level A = Most likely a serious problem - resolve or explain
  1 ALERT level B = A potentially serious problem, consider carefully
  7 ALERT level C = Check. Ensure it is not caused by an omission or oversight
 26 ALERT level G = General information/check it is not something unexpected

  6 ALERT type 1 CIF construction/syntax error, inconsistent or missing data
 20 ALERT type 2 Indicator that the structure model may be wrong or deficient
  5 ALERT type 3 Indicator that the structure quality may be low
  1 ALERT type 4 Improvement, methodology, query or suggestion
  3 ALERT type 5 Informative message, check
```

It is advisable to attempt to resolve as many as possible of the alerts in all categories. Often the minor alerts point to easily fixed oversights, errors and omissions in your CIF or refinement strategy, so attention to these fine details can be worthwhile. In order to resolve some of the more serious problems it may be necessary to carry out additional measurements or structure refinements. However, the purpose of your study may justify the reported deviations and the more serious of these should normally be commented upon in the discussion or experimental section of a paper or in the "special_details" fields of the CIF. checkCIF was carefully designed to identify outliers and unusual parameters, but every test has its limitations and alerts that are not important in a particular case may appear. Conversely, the absence of alerts does not guarantee there are no aspects of the results needing attention. It is up to the individual to critically assess their own results and, if necessary, seek expert advice.

Publication of your CIF in IUCr journals

A basic structural check has been run on your CIF. These basic checks will be run on all CIFs submitted for publication in IUCr journals (*Acta Crystallographica*, *Journal of Applied Crystallography*, *Journal of Synchrotron Radiation*); however, if you intend to submit to *Acta Crystallographica Section C* or *E* or *IUCrData*, you should make sure that full publication checks are run on the final version of your CIF prior to submission.

Publication of your CIF in other journals

Please refer to the *Notes for Authors* of the relevant journal for any special instructions relating to CIF submission.

PLATON version of 30/01/2018; check.def file version of 30/01/2018

Datablock Coesite-IV_44.18GPa - ellipsoid plot

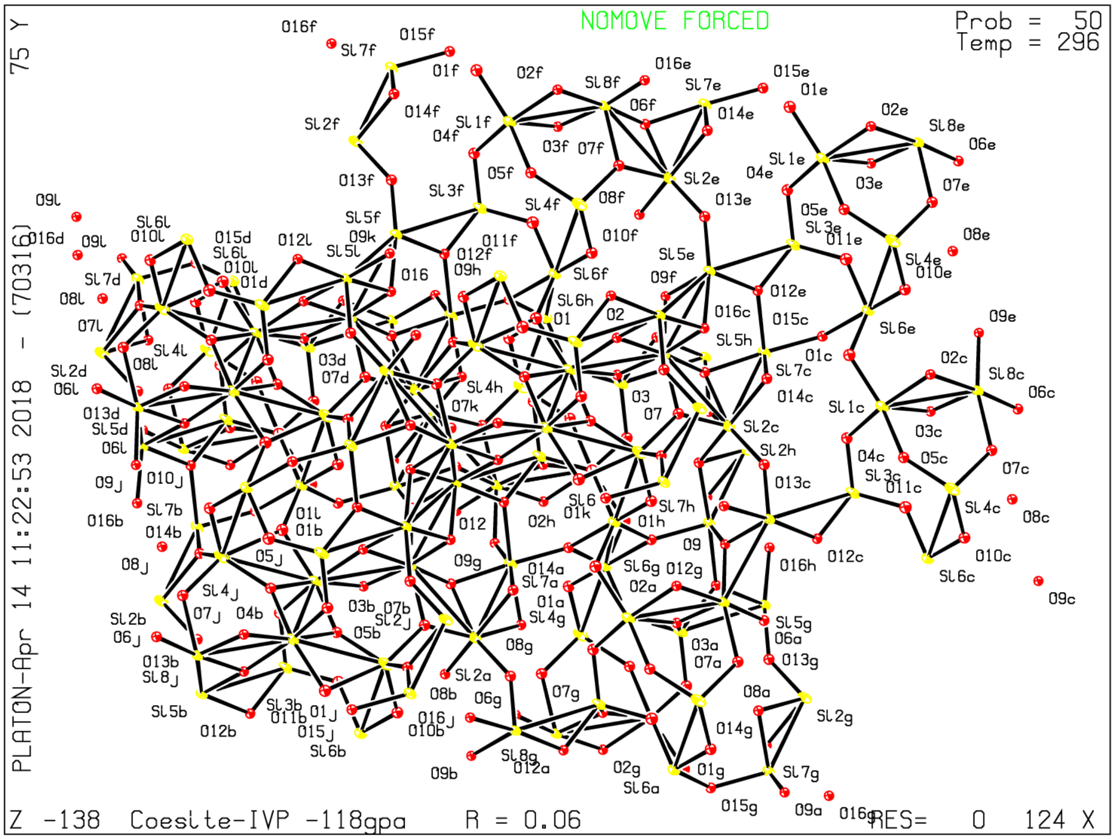

Supplement: Supplementary file 13 — Supplementary Data 10 [file 41467_2018_7265_MOESM13_ESM.pdf]
